# Mitochondrial Alterations in PINK1 Deficient Cells Are Influenced by Calcineurin-Dependent Dephosphorylation of Dynamin-Related Protein 1

**DOI:** 10.1371/journal.pone.0005701

**Published:** 2009-05-27

**Authors:** Anna Sandebring, Kelly Jean Thomas, Alexandra Beilina, Marcel van der Brug, Megan M. Cleland, Rili Ahmad, David W. Miller, Ibardo Zambrano, Richard F. Cowburn, Homira Behbahani, Angel Cedazo-Mínguez, Mark R. Cookson

**Affiliations:** 1 Laboratory of Neurogenetics, National Institute on Aging, Bethesda, Maryland, United States of America; 2 Alzheimer's Disease Research Center, Karolinska Institutet, Stockholm, Sweden; 3 Surgical Neurology Branch, National Institute of Neurological Diseases and Stroke, Bethesda, Maryland, United States of America; Emory University, United States of America

## Abstract

PTEN-induced novel kinase 1 (PINK1) mutations are associated with autosomal recessive parkinsonism. Previous studies have shown that PINK1 influences both mitochondrial function and morphology although it is not clearly established which of these are primary events and which are secondary. Here, we describe a novel mechanism linking mitochondrial dysfunction and alterations in mitochondrial morphology related to PINK1. Cell lines were generated by stably transducing human dopaminergic M17 cells with lentiviral constructs that increased or knocked down PINK1. As in previous studies, PINK1 deficient cells have lower mitochondrial membrane potential and are more sensitive to the toxic effects of mitochondrial complex I inhibitors. We also show that wild-type PINK1, but not recessive mutant or kinase dead versions, protects against rotenone-induced mitochondrial fragmentation whereas PINK1 deficient cells show lower mitochondrial connectivity. Expression of dynamin-related protein 1 (Drp1) exaggerates PINK1 deficiency phenotypes and Drp1 RNAi rescues them. We also show that Drp1 is dephosphorylated in PINK1 deficient cells due to activation of the calcium-dependent phosphatase calcineurin. Accordingly, the calcineurin inhibitor FK506 blocks both Drp1 dephosphorylation and loss of mitochondrial integrity in PINK1 deficient cells but does not fully rescue mitochondrial membrane potential. We propose that alterations in mitochondrial connectivity in this system are secondary to functional effects on mitochondrial membrane potential.

## Introduction

Several inherited diseases have been identified that share the core clinical characteristics of rigidity, bradykinesia and resting tremor with Parkinson's disease (PD) [1]. These symptoms largely result from dopaminergic cell loss in the *substantia nigra*. Mitochondria have been proposed to play a key role in the pathogenesis of PD as, for example, mitochondrial toxins are used to generate animal models of dopaminergic cell loss [2].

Three genes associated with recessive parkinsonism in humans, *parkin*, *DJ-1* and *PTEN induced kinase 1* (*PINK1*), have all been shown to be involved in mitochondrial function and protection against oxidative stress. DJ-1 protects against oxidative stress and localizes in part to mitochondria [3]. Parkin is an E3 ubiquitin ligase that may play an as yet undefined role in mitochondrial function [4], [5]. Parkin may associate with the outer mitochondrial membrane in order to prevent mitochondrial swelling and cell death [6] and recently has been shown to promote the degradation of depolarized mitochondria by autophagy [7].

Studies in *Drosophila* have shown that the cytosolic E3 ubiquitin ligase parkin and the mitochondrial kinase PINK1 are involved in a pathway that maintains mitochondrial morphology where PINK1 is active upstream of parkin [8]–[10]. Supporting this observation, fibroblasts from patients homozygous for G309D PINK1 mutation have mitochondrial dysfunction, increased lipid peroxidation and elevated antioxidant defense in mitochondrial superoxide dismutase and glutathione [11]. These changes alter mitochondrial morphology and are exaggerated by manipulation of glucose levels *in vitro*, which stress the cells [12]. Similar effects are seen in parkin deficient fibroblasts, with impaired complex I activity and mitochondrial morphology changes [13]. Two independent groups have shown that PINK1 deficient neuronal cells have defects including mitochondrial fragmentation, swelling and cristae abnormalities that increase over time [14], [15]. Some of these defects appear to be related to a poor ability of PINK1 deficient mitochondria to buffer calcium [16], which in turn may be related to a decreased turnover of impaired mitochondria via mitophagy [15]. PINK1 knockout mice have no mitochondrial morphological defects [17] but their mitochondria are functionally impaired [18], as are mitochondria in cell lines where PINK1 is knocked down [19], [20].

The above literature suggests that loss of PINK1 can be associated with functional and morphological effects on mitochondria. The overall morphology of mitochondria is influenced by a number of mechanisms, including the relative rates of mitochondrial fission and fusion. Both mitochondrial fission and fusion are highly regulated processes that are critical for the function of mitochondria and may be especially important in neurons [21]. Several recent results suggest that PINK1 may alter the balance between fusion and fission, although there is some controversy about how exactly PINK1 may function. Increased expression of proteins that promote fission, namely dynamin-related protein 1 (Drp1), rescues PINK1 deficient phenotypes in *Drosophila* models [22], [23]. In contrast, PINK1 prevents rather than promotes fission in *C elegans* [24]. Recent studies in mammalian cells suggest that dominant negative Drp1 constructs antagonize the effects of loss of PINK1 [15] and the morphological effects of PINK1 deficiency are more closely associated with mitochondrial fission rather than fusion [12], [15], although Yang *et al* reported a fusion-like phenotype in COS7 cells [23] and another study reported no morphological effects related to lack of PINK1 [20]. The fact that PINK1 protects against rotenone-induced damage, which is reported to be associated with mitochondrial fission in mammalian cells [25], indirectly suggests that PINK1 might antagonize pro-fission processes. Table 1 lists some of the previous studies regarding the role of PINK1 in the balance of mitochondrial fission and fusion. The first aim of the current study was to therefore measure mitochondrial connectivity directly in living human dopaminergic neuroblastoma cells to attempt to resolve whether the phenotype relates to mitochondrial fission or fusion.

**Table 1:** Effects of mitochondrial fusion and fission proteins on PINK1 phenotypes in different models.

Reference	System	Fission/Fusion manipulation	Effect	Interpretation
Poole et al [22]	Drosophila Genomic knockout	Drp1 LOF (heterozygote)	Lethality	
		Drp1 expression or Opa1/Mfn2 LOF	Suppresses PINK1 LOF phenotypes	PINK1 promotes fission
Yang et al [23]	Drosophila Genomic knockout	Drp1 LOF (heterozygote)	Enhances PINK1 LOF phenotypes	
		Drp1 expression or Opa1/Mfn2 LOF	Suppresses PINK1 LOF phenotypes	
	COS7 (African green monkey) cells, transient siRNA	Fis1 expression or Drp1 expression	Suppresses PINK1 LOF phenotypes	PINK1 promotes fission
Deng et al. [36]	Drosophila Genomic knockout	Drp1 LOF (heterozygote)	Lethality	
		Drp1 expression or Opa1/Mfn2 LOF	Suppresses PINK1 LOF phenotypes	PINK1 promotes fission/limits fusion
Dagda et al. [15]	SY5Y (Human) cells, stable siRNA	Drp1-dominant negative expression	Suppresses PINK1 phenotypes	PINK1 limits fission
This study	SY5Y (Human) cells, stable siRNA	Drp1 expression	Additive to PINK1 LOF phenotypes	
		Drp1-knockdown, Opa1/Mfn2 expression	Suppresses PINK1 phenotypes	PINK1 limits fission or promotes fusion

Prior studies are listed where the effects of manipulating the expression or activity of fission and/or fusion proteins was measured against PINK1 loss of function mutations. LOF, loss of function.

Mechanism(s) involved in PINK1-dependent maintenance of morphology and cell viability are not currently well defined [26]. As PINK1 is a kinase, it is likely that there are key substrate(s) that affect mitochondrial morphology. One possible PINK1 substrate, TRAP1 [27], is mitochondrial. PINK1 has also been proposed to modulate the phosphorylation status of another mitochondrial protein Omi/HtrA2, possibly through indirect mechanisms [28]. It is not clear if these substrates contribute to the morphological effects of loss of PINK1. Therefore, the second aim of the current study was to address the underlying mechanism by which PINK1 influences mitochondrial morphology.

Using live cell imaging, we show that overexpression of PINK1 protects against loss of connectivity induced by rotenone in a kinase-dependent fashion and also that PINK1 deficiency induces mitochondrial fragmentation. We show that the activity of the fission GTPase Drp1, which is controlled by the phosphatase calcineurin, contributes to the effects of PINK1 on mitochondrial morphology.

## Results

### PINK1 influences mitochondrial function

**Figure 1:**
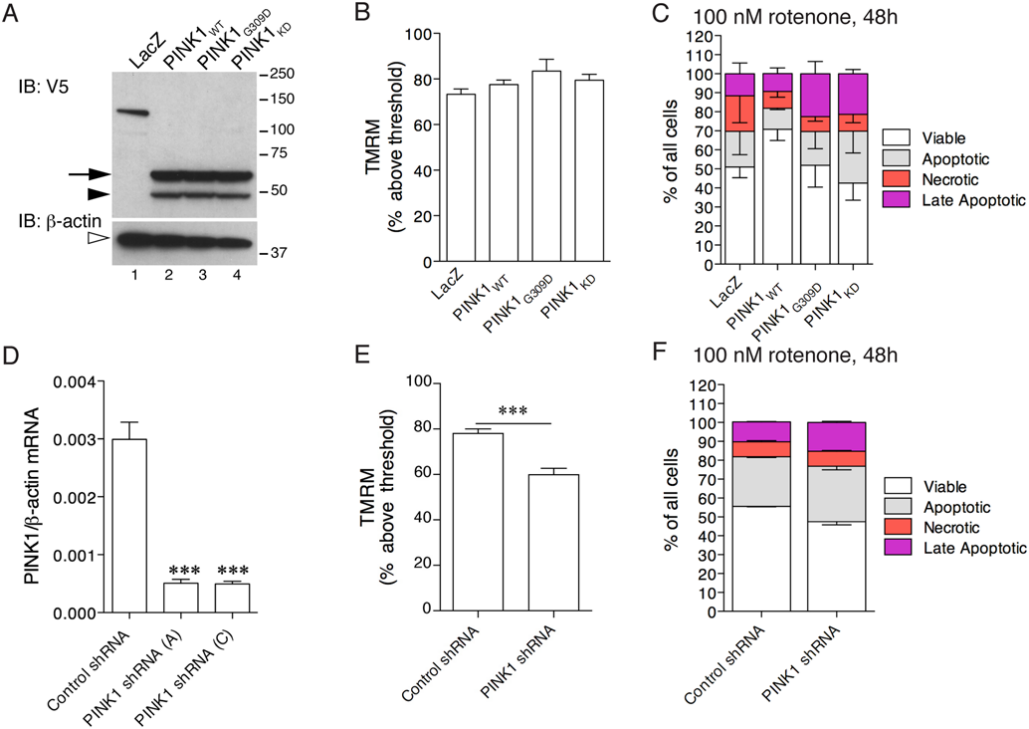
PINK1 expression alters mitochondrial function and cellular viability. (A–C) Stable overexpression of PINK1 variants. (A) Western blot of stable cell lines generated using lentiviral transduction and selected for equal expression of PINK1. A control lentivirus expressing LacZ is shown in lane 1, lanes 2–4 are cell lines expressing wild-type (WT), G309D or kinase dead (KD) PINK1. The PINK1 in these cells is V5-tagged at the C-terminus, the precursor (arrow) and mature (closed arrowhead) forms of PINK1 are visible. In the lower panel, the blot was reprobed with β-actin to show equal loading. Molecular weight markers on the right are in kilodaltons. (B) Mitochondrial membrane potential was measured using FACS in live cells using TMRM. Results are expressed as the mean number of cells with TMRM fluorescence above threshold set by depolarizing one set of cells with CCCP (100 µM, 10 minutes; see Supplementary Fig. S1). Error bars show the SEM (n = 6–7 independent experiments per line). There are no statistically significant differences between the lines (*P* = 0.23 by ANOVA). (C) Cell viability was measured after exposure of cells to 100 nM rotenone for 48 hours using FACS. Viable cells were defined as AnnexinV (AnnV) and propidium iodide (PI) negative, and are expressed as percentage of all sorted cells; apoptotic cells were AnnV positive/PI negative; necrotic cells were AnnV negative/PI positive; late apoptotic cells were AnnVpositive/PI positive. Each bar is the mean of 3 experiments with 10,000 cells counted in each, and error bars indicate the SEM between experiments. The improvement in cell viability was significant for cells expressing WT PINK1 only (*p* = 0.03). (D–F) Stable knockdown of PINK1. (D) Bars show the mean relative PINK1 expression estimated using quantitative RT-PCR, normalized to β-actin expression. Two different PINK1 shRNA sequences, A and C, decrease endogenous mRNA expression relative to a non-specific control shRNA. The differences between the cell lines were assessed using one-way ANOVA (*P*<0.0001 overall) and Dunnett's multiple comparison *post hoc* tests when compared to the control shRNA cell line; ***, P<0.0001 (n = 6 independent experiments, error bars indicate the SEM). (E) Mitochondrial membrane potential was measured and is expressed as in (B). The differences between control and PINK1 shRNA were significant by *t*-test (*P* = 0.0008, *n* = 5 independent experiments). (F) Cell viability after rotenone exposure, as in (C), was lower in PINK1 deficient cells compared to control shRNA. The difference in the percentage of viable cells was significant (*P* = 0.009 by t-test, *n* = 3).

To address the function of PINK1 in living cells, we generated stable dopaminergic neuroblastoma lines that express wild-type PINK1, a recessive mutant, G309D and an artificial variant lacking kinase activity [29] (Fig. 1A–C). Because PINK1 mutations are recessive, we also examined cells stably transduced with either of two shRNA sequences directed against PINK1, with a scrambled shRNA used as control (Fig. 1D–F).

PINK1 overexpression lines had equivalent expression of protein, assessed using western blotting (Fig. 1A). We also performed immunocytochemistry for the V5 tag, which demonstrated that all cells in each clone expressed V5-PINK1, and RT-PCR, which showed that the exogenous constructs were expressed at approximately 4-fold over endogenous PINK1 (data not shown). No available antibodies screened to date are able to detect endogenous PINK1 without having additional protein bands (data not shown), so qRT-PCR was used to confirm decreased PINK1 expression in the shRNA lines. Expression was decreased by >80% with the first sequence (A) and by a similar amount with a second shRNA (C) (Fig. 1D).

PINK1 can protect against rotenone toxicity [30], [31] and is important in maintaining mitochondrial membrane potential (Δψ_m_) [12], [14] in mammalian cells. We used these observations to confirm that our PINK1 constructs behaved as expected. Cells expressing PINK1 maintained Δψ_m_ estimated using TMRM (Fig. 1B), similar to previous studies [31], [32]. In contrast, cells transduced with a PINK1 shRNA lentivirus showed decreased Δψ_m_ (Fig. 1E; *P* = 0.0031 for cell lines), again consistent with recent reports [12]. Cells expressing PINK1 also showed improved viability after exposure to 100 nM rotenone (two-way ANOVA; *P* = 0.0004 for cell lines; *P* = 0.0001 for drug effects) (Fig. 1C). Although the increase in net viability is modest (∼20%), this is similar to the effect of PINK1 overexpression in previous studies [10], [30], [33]. Alternative assays for cell viability confirmed that PINK1 partially protects against rotenone toxicity (Supplementary Fig. S2A). We also examined cell viability after rotenone exposure in PINK1 deficient cells and their appropriate control shRNA line (Fig. 1F). The control shRNA line showed a similar amount of decrease in viability after rotenone exposure (55.64+/−0.25%, *n* = 3 experiments with 10,000 cells analyzed/experiment) as the LacZ control for the overexpression (51.0+/−5.6% *n* = 3), suggesting a lack of off-target effects of the shRNA. In contrast, PINK1 shRNA cells showed a greater loss of viability after rotenone exposure, to 47.4+/−1.71% of cells being viable (*P* = 0.009 by t-test comparing the proportion of viable cells in control shRNA with PINK1 shRNA lines). These results suggest that PINK1, via its kinase activity, has beneficial effects on mitochondrial function under stressed conditions that improve cell viability.

The above results show that our PINK1 lentiviral constructs have the expected effects in living cells. One small point of difference with the literature [14], [30], [31] is that in these cell lines, PINK1 does not protect against staurosporine-induced apoptosis (Supplementary Fig. S2D). With rotenone exposure, events typically associated with apoptotic cell death such as Bax multimerization and PARP cleavage are not observed, but are seen following staurosporine treatment (Supplementary Fig. S2E, F). FACS analyses showed both apoptosis and necrosis occurred in our model (Fig. 1C, F). These results suggest that the effect of PINK1 is not on apoptosis *per se* but on mitochondrial function.

**Figure 2:**
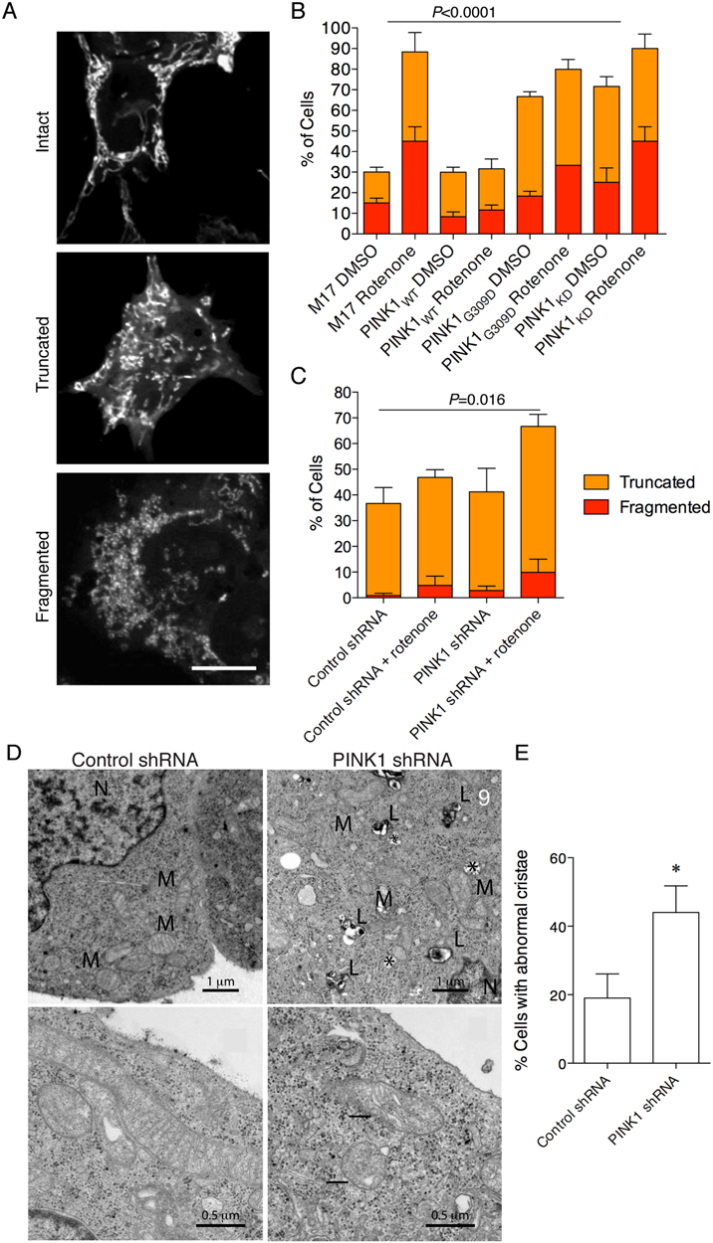
PINK1 influences mitochondrial morphology in response to rotenone. (A) M17 cell lines were stained with mitotracker and cells assigned to one of three morphological categories; intact, truncated or fragmented. Scale bar in the bottom panel is 10 µm, applies to all fluorescence micrographs. (B, C) Blinded counts of 45–130 cells in each of three experiments were performed in cell lines overexpressing PINK1 (B) or lacking PINK1 (C) with or without exposure to 100 nM rotenone for 24 hours. Cells with truncated or fragmented mitochondria are expressed as a percentage of the total counted. Error bars indicate the SEM between experiments. Statistical significance was assessed by two-way ANOVA for mitochondrial morphology and cell line. *P* values for cell groups were significant and are shown above each figure. (D) Transmission electron microscope images of control shRNA or PINK1 shRNA lines. At low magnification (upper panels), an association of mitochondria (M) with lysosomes (L) is seen, particularly in the PINK1 deficient cells. Occasional vacuoles are also seen, indicated by asterisks. N = nucleus. At higher power (lower panels), the mitochondrial cristae in PINK1 deficient cells are disrupted (arrows) while normal mitochondrial morphology is seen in control cells. Scale bars are shown for each figure and are 1 µm in the top panels and 0.5 µm in the lower panels. (E) Counting abnormal cristae across multiple experiments shows that approximately twice as many cells in the PINK1 deficient lines showed mitochondrial cristae abnormalities compared to the control shRNA (*, *P* = 0.032 by t-test, *n* = 8).

Next we examined mitochondrial morphology in these knockdown cell lines using the approach outlined by Exner et al [12], who divided the mitochondrial morphologies of PINK1 deficient cells into intact, truncated and fragmented categories (Fig. 2A). Rotenone was used as a stress-inducing agent, which has been shown to cause mitochondrial truncation and fragmentation in various cell types [25], [34], [35]. Increased expression of wild type PINK1 limited the number of cells with fragmented/truncated mitochondria after exposure to rotenone (Fig. 2B; *P*<0.0001 overall for cell lines/treatments by two-way ANOVA). PINK1 knockdown increased the number of cells containing truncated and fragmented mitochondria, and rotenone had an additive effect (Fig. 2C; *P* = 0.016 for differences between cell lines/treatments). It should be noted that the number of cells showing a full fission phenotype was low (10–20% of total) and so the effect of PINK1 deficiency on fission may be indirect, perhaps suggesting a general effect on mitochondria that can result in a number of aberrant morphologies. Using transmission electron microscopy (TEM; Fig. 2D), we saw swollen cristae and a possible association of mitochondria with lysosomes in PINK1 deficient cells, similar to previous reports [14], [15]. We counted that 44+/−7.8% of cells had mitochondria containing swollen cristae in the PINK1 deficient cells, which was significantly (Fig. 2E; *P* = 0.032 by t-test) higher than in control shRNA lines (19+/−7.1%). Collectively, these results show that PINK1 has subtle but measureable effects on mitochondrial morphology.

### PINK1 limits mitochondrial fragmentation after rotenone exposure

**Figure 3:**
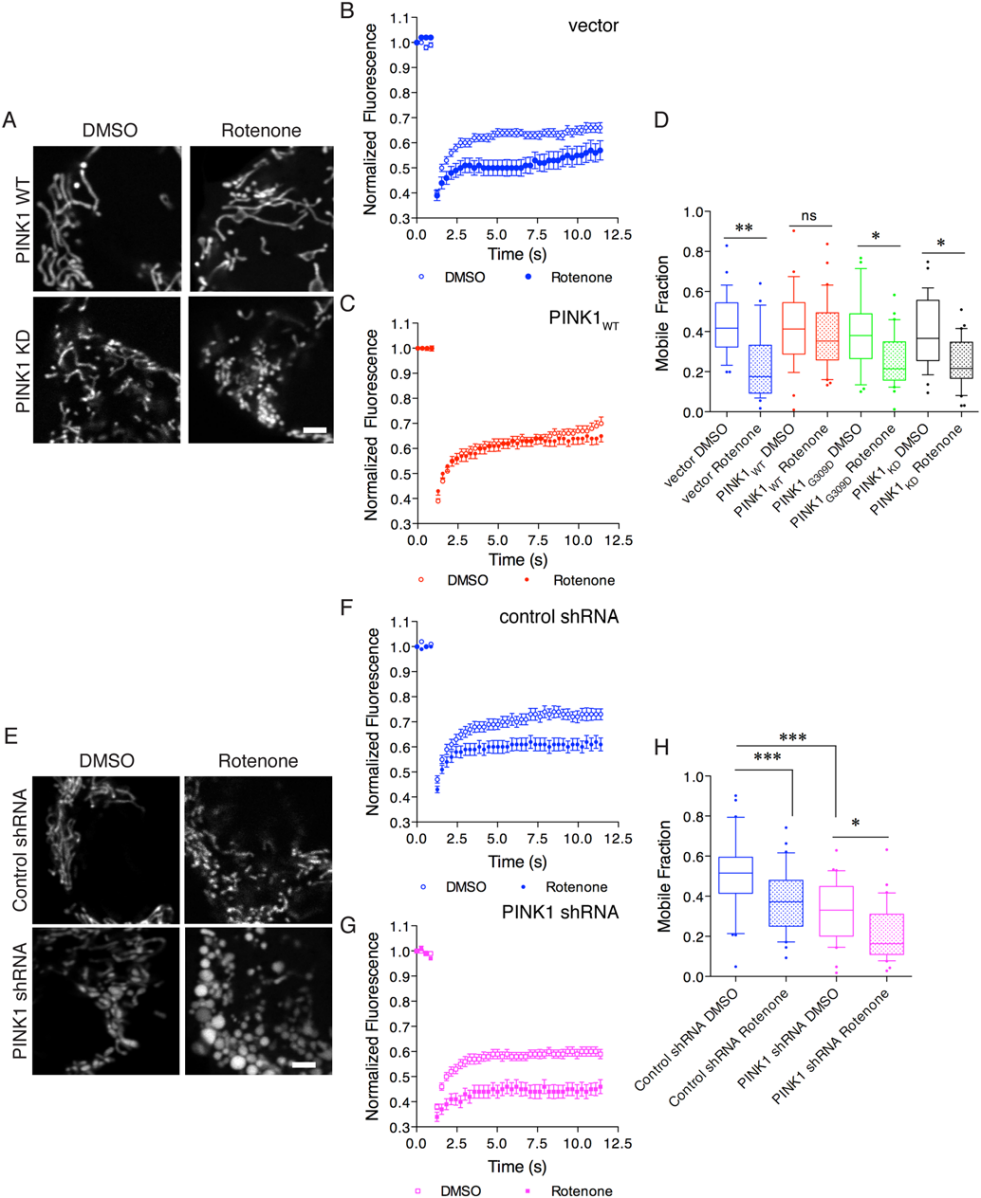
PINK1 protects against rotenone-induced loss of mitochondrial connectivity. (A) Living cells were imaged after transfection with mito-YFP. Cells expressing PINK1 had longer mitochondria compared to control lines (kinase dead shown here). Rotenone induced fragmentation in control lines but not in lines expressing PINK1. Scale bar in the lower right panel is 2 µm, applies to all fluorescence micrographs. (B) FRAP curves demonstrate that control cells showed more recovery under control conditions (open circles) than after 24 h exposure to 100 nM rotenone (closed circles). In this experiment, control cells are parental M17 cells that were transfected only with the mito-YFP vector to image mitochondria (labeled vector). (C) In contrast, cells stably transduced with wild-type PINK1 showed very similar FRAP curves whether this was measured in the presence of rotenone (closed circles) or under control conditions (open circles, DMSO was used as a vehicle for rotenone). Cells transfected with G309D or kinase dead PINK1 also responded to rotenone (data not shown). In B and C, each point is the average of >30 separate measurements and is representative of at least triplicate experiments for each line/treatment. Error bars indicate the SEM. (D) Mobile fraction of mito-YFP was measured in the indicated cell lines (colors as in B, filled boxes are rotenone treated). In this and all other plots of mobile fraction, summaries are of 24–30 cells. The box indicates the upper and lower quartiles, central line indicates the median and range bars indicate the 10^th^ to 90^th^ percentile range. Cells lying outside of this range are shown as single points. Differences between treatments were significant overall (*P*<0.0001 by ANOVA) and Student-Newman-Kuells' *post-hoc* test was used to evaluate rotenone effects in each line. *P<0.05; ** *P*<0.01; ***P<0.001; ns = not significant (*P*>0.05). (E) Cells expressing an shRNA against PINK1 showed some basal evidence of fission, with some fragmented mitochondria visible, and exaggerated responses to rotenone and mitochondrial swelling. Scale bar in the lower right panel is 2 µm, applies to all fluorescence micrographs. (F–H) FRAP analyses as (in B,C) showing control shRNA lines (F) and shRNA against PINK1 (G). Open symbols are untreated cells, closed symbols were treated with 100 nM rotenone for 24 hours. (H) Mobile Fraction values were calculated from FRAP curves as in (D). Differences between treatments were significant overall (*P*<0.0001 by ANOVA; *n* = 30 cells, representative of at least three experiments per cell line) and Student-Newman Kuells' *post-hoc* test was used to evaluate rotenone effects in each line. *P<0.05; ** *P*<0.01; ***P<0.001; ns = not significant (*P*>0.05). Additional shRNA sequences are shown in Supplementary Figure S2.

To provide a more quantitative estimate of functional mitochondrial connectivity, we used fluorescence recovery after photobleaching (FRAP) in living cells. Using mito-YFP to label mitochondria, cells expressing wild-type, but not kinase dead, PINK1 maintained connected mitochondrial morphology in the presence of rotenone (Fig. 3A). Under control conditions, we saw a loss of fluorescence upon photobleaching that partially recovered over time (Fig. 3B). Rotenone treatment in control M17 cell lines transfected with the mito-YFP vector alone (vector in Fig. 3B) significantly lowered the recovery of fluorescence, consistent with loss of connectivity in the mitochondrial network as reported previously [25], [34], [35]. The same effect was seen in cell lines expressing recessive G309D or kinase dead PINK1 (data not shown). In contrast, FRAP in cells expressing wild-type PINK1 was similar with or without rotenone treatment, showing that PINK1 functionally protects against rotenone-induced mitochondrial fragmentation (Fig. 3C). We calculated the mobile fraction of mito-YFP from the FRAP data, which confirmed that PINK1 protects against loss of connectivity in the mitochondrial network in a kinase-dependent fashion (Fig. 3D). The difference between rotenone treated and untreated cells was significant for control M17 (*P*<0.01), G309D and kinase dead PINK1 stable lines (*P*<0.05) but not for wild-type PINK1. A similar protection against rotenone-induced loss of mitochondrial connectivity was seen when wild-type PINK1 was transiently transfected into naïve M17 cells (data not shown), arguing against clonal effects.

To confirm that this effect is seen with endogenous PINK1, we examined cells stably expressing PINK1 shRNA, in this case using a scrambled shRNA sequence as a control (Fig. 3E–H). PINK1 knockdown cells had a partially fragmented and swollen morphology and a lower FRAP compared to control shRNA cells. After rotenone exposure, mitochondria were swollen and lost connectivity, particularly in the absence of PINK1 (Fig. 3E–G). We confirmed the effect in separate experiments with an independent shRNA sequence and a different shRNA control cell line (Supplementary Fig. S3), thus excluding a non-specific shRNA effect. We also compared the control shRNA line to parental M17 cells and did not see a statistically significant effect between the two but found that the PINK1 deficient cells had significantly lower mobile fraction values when compared to either cell line (Supplementary Fig. S3C).

**Figure 4:**
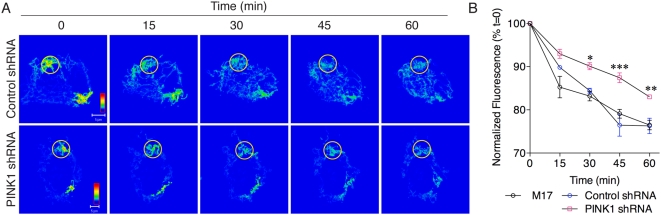
PINK1 deficient cells have fewer mitochondrial fusion events. (A) Control shRNA (upper panels) or PINK1 shRNA (lower panels) cells were transfected with a mitochondrially directed, photoactivatable GFP which was then photoactivated within a small region of interest (see Materials and Methods). Sequential images of the single cells (0, 15, 30, 45 and 60 minutes as indicated above the images) after photoactivation are shown. When cells are fusion-competent, decreases in fluorescence are seen over time in the region of photoactivation. Note that the fluorescence intensity is equal across the cell in the control line in the upper panel by 30 minutes, whereas the PINK1 deficient cell retains areas of higher intensity, indicating decreased fusion. Each scale bar is 5 µm and applies to all fluorescence micrograph in that series. (B) Quantification of experiments as in A (N = 3 experiments, with n = 9–10 cells measured per time point per experiment) shows the loss of fluorescence over time for parental M17 cells (black triangles), control shRNA (blue circles) or PINK1 shRNA (pink squares) cell lines. The difference between cell lines was significant by two-way ANOVA (P<0.001), as was the effect of time (P<0.001). Bonferonni *post-hoc* tests were used to compared PINK1shRNA lines to the control shRNA lines *P<0.05; ** *P*<0.01; ***P<0.001. Error bars indicate the SEM between experiments.

One possible interpretation of previous studies of loss of PINK1 in *Drosophila* is that there is an increased rate of mitochondrial fusion [22], [23], [36]. To address this directly, we estimated mitochondrial fusion in control and PINK1 deficient cells using photoactivatable GFP, where fusion results in a loss of GFP fluorescence over time. Both parental M17 and control shRNA lines showed a similar loss of pA-GFP fluorescence when quantified over time whereas PINK1 deficient cells had a slower loss of pA-GFP (Fig. 4A, B). Using two-way ANOVA, the differences in pA-GFP signal were significantly different over time, as expected (*P*<0.001) but also differed significantly between lines (*P*<0.001; see Fig. 4B for post-hoc comparisons between cell lines at each time point). Therefore, knockdown of PINK1 does not result in an increase but, instead, a slight decrease in the apparent rate of mitochondrial fusion.

### Effects of Drp1 on mitochondrial connectivity

**Figure 5:**
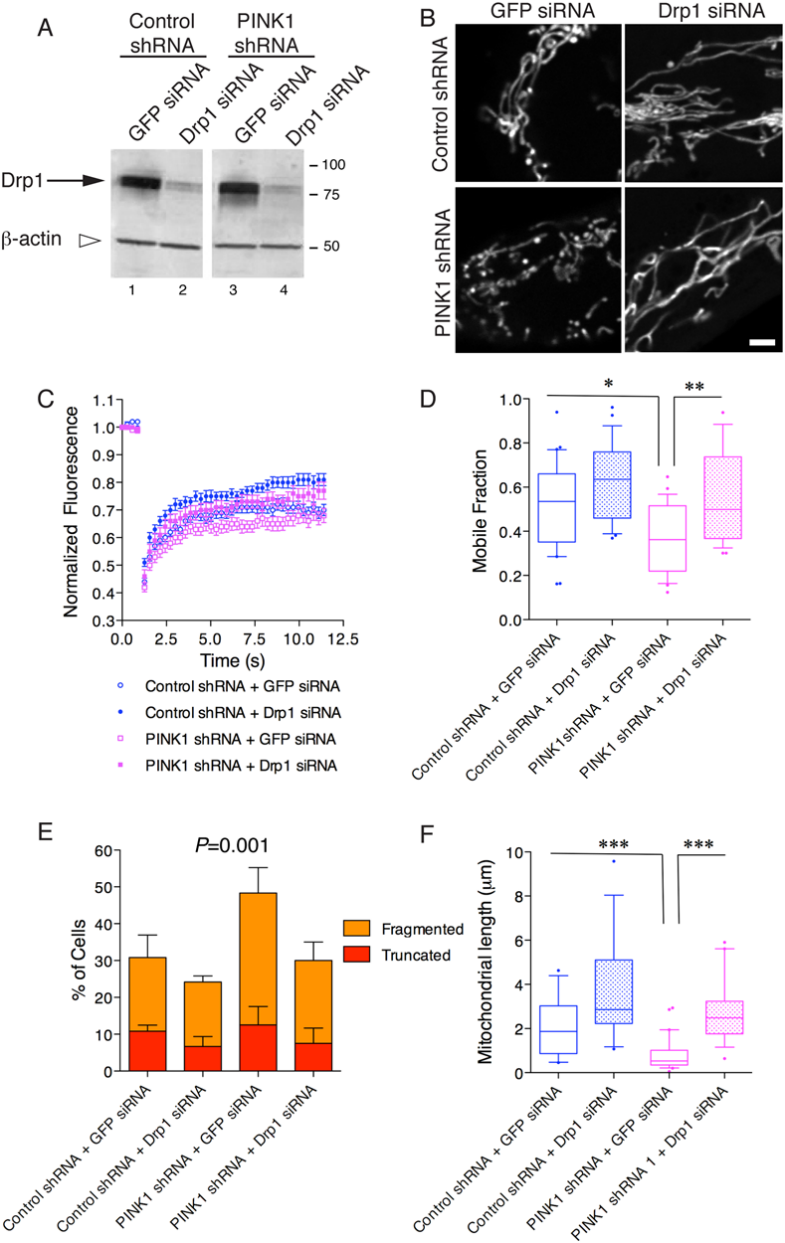
Mitochondrial effects due to loss of PINK1 can be rescued by knockdown of Drp1. (A) Exposure of control shRNA or PINK1 shRNA stable cell lines to Drp1 siRNA for four days results in an approximately 70% knockdown of Drp1 (arrow) compared to a non-specific GFP siRNA. β-actin shows equal loading. Molecular weight markers are in kilodaltons. (B) Live cell images of mito-YFP transfected control shRNA (upper panels) and PINK1 shRNA (lower panel) cells exposed to a non-specific siRNA against GFP (left panels) or an siRNA against Drp1 (right panels). Scale bar is 2 µm, applies to all fluorescence micrographs. (C) FRAP curves show that under basal situations, there was a difference between the control shRNA line and the PINK1 shRNA line. These differences were partially normalized after the cells were exposed to an siRNA against Drp1. Each point is the average of >30 separate measurements and error bars indicate the SEM. (D) Mobile fraction of mito-YFP was measured in control shRNA or PINK1 shRNA cell lines either after expression of a control siRNA (against GFP) or after expression of an siRNA against Drp1. Boxplots summarize data from *n* = 24–30 cells and are representative of duplicate experiments. Differences between treatments were significant overall (*P*<0.0001 by ANOVA) and Student-Newman Kuells' *post-hoc* test was used to evaluate the differences between control shRNA and PINK1 shRNA after each treatment. *P<0.05; ** *P*<0.01. (E) Counts of mitochondrial morphology as in figure 2 were performed on n = 60 cells from duplicate experiments. Drp1 siRNA decreased the number of cells with truncated or fragmented mitochondria. Differences were analyzed by two-way ANOVA using morphology and cell line/treatment as factors, *P* = 0.001 overall for the different cell groups. (F) Mitochondrial length was measured from images of unfixed, mito-YFP transfected cells. Boxes indicate the upper and lower quartiles, central line indicates the median and range bars indicate the 10^th^ to 90^th^ percentile range. Differences between treatments were significant overall (*P*<0.0001 by ANOVA, *n* = 14–29) and Student-Newman Kuells' *post-hoc* test was used to evaluate the differences between control shRNA and PINK1 shRNA after each treatment. *** *P*<0.001.

We asked whether loss of Dynamin related protein 1 (Drp1), a GTPase involved in various aspects of mitochondrial morphology and function, would antagonize the effects of PINK1 deficiency. Using siRNA to Drp1, we achieved knockdown to ∼30% of controls and is similar in control or PINK1 shRNA lines (Fig. 5A). This partial knockdown resulted in an elongated mitochondrial network in both cell lines (Fig. 5B), similar to previous studies in other cell types [37]–[39]. This partial knockdown prevented mitochondrial fragmentation associated with loss of PINK1 expression in the absence of other stressors. Calculating the mobile fraction of mitoYFP, the difference between control shRNA and PINK1 shRNA lines remained significant (*P*<0.05 by one-way ANOVA with Student-Newmann Kuell's *post-hoc* test) after expression of a control siRNA directed against GFP. The mobile fraction in PINK1 shRNA cells was significantly different after Drp1 RNAi compared to GFP RNAi (*P*<0.01; Fig. 5D). A similar rescue of the mobile fraction deficit was seen in cell lines expressing the second PINK1 shRNA sequence (data not shown). Cell counts show a decrease in the proportion of cells with fragmented or truncated mitochondria after Drp siRNA (*P* = 0.001 by two-way ANOVA; Fig. 5E). Finally, mitochondrial length was measured and was lower in PINK1 deficient cells compared to control (*P*<0.001 by one-way ANOVA) and increased after Drp siRNA (*P*<0.001; Fig. 5F).

**Figure 6:**
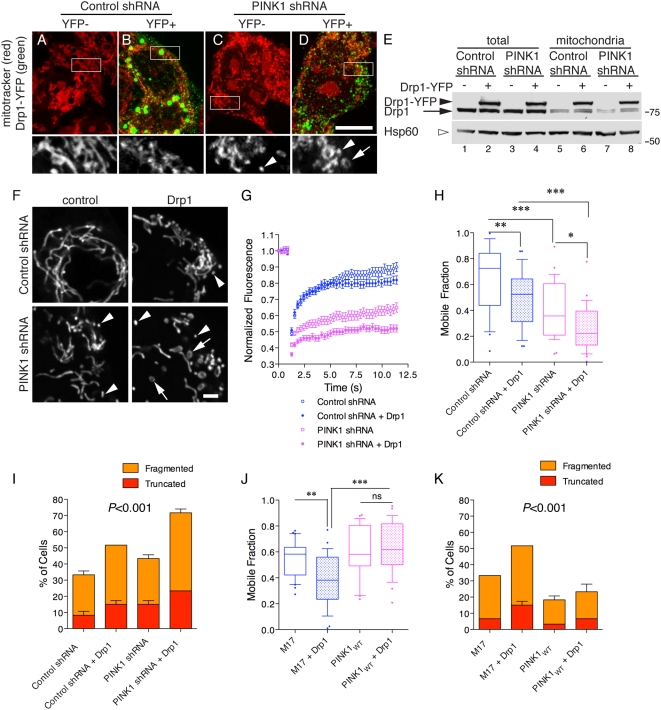
PINK1 deficiency and increased Drp1 expression have additive effects. (A–D) Control shRNA (A,B) or PINK1 shRNA (C,D) were transfected with YFP-Drp1 (green in upper panels) and stained with mitotracker (red in upper panels; lower panels show an enlarged portion of the mitotracker channel only). At 24 hours after transfection, PINK1 shRNA lines (D) showed more mitochondrial damage than control shRNA lines. Arrowheads show small fragments of mitochondria that are particularly seen in PINK1 deficient cells, arrows show circular fragments that are only seen in PINK1 deficient cells. Scale bar in the lower panel of (D) is 10 µm and applies to all photomicrographs. (E) Control shRNA and PINK1 shRNA cells were transfected with YFP or YFP-Drp1. Total cell lysates or mitochondrial fractions were blotted for Drp1 or Hsp60 as a loading control. Markers on the right are in kilodaltons. (F) Control shRNA (upper panels) or PINK1 shRNA cells (lower panels) were transfected with mito-YFP without (left panels) or with Drp1 (right panels) and live images taken. As in A–D, arrowheads show small fragments and arrows circular remnants of mitochondria. Scale bar in the lower right panel is 2 µm, applies to all fluorescence micrographs. (G, H) FRAP was used to assess the effect of Drp1 overexpression as in figure 3. Open symbols are untransfected cells, closed symbols were transfected with Drp1 for 24 hours. The FRAP curves over time in (G) are the average of 30 observations, representative of duplicate experiments. Error bars indicate the SEM. (H) Summary data for the mobile fractions are shown from *n* = 30 cells, representative of duplicate experiments. Differences between treatments were significant overall (*P*<0.0001 by ANOVA) and Student-Newman Kuells' *post-hoc* test was used to compare each line with and without Drp1 expression. *P<0.05; ** *P*<0.01; ***P<0.001. (I) Counts of mitochondrial morphology as in figure 2 were performed on 60 cells from duplicate experiments in control shRNA or PINK1 shRNA cell lines. Differences were analyzed by two-way ANOVA using morphology and cell line/treatment as factors and *P* values for cell groups are given above each graph. (J) M17 or stable PINK1 WT cells were transfected with Drp1. FRAP curves (data not shown) were generated and mobile fractions were derived. (J) Differences between treatments were significant overall (*P*<0.0001 by ANOVA, *n* = 30 cells) and Student-Newman Kuells' *post-hoc* test was used to compare each line with and without Drp1 expression. *P<0.05; ** *P*<0.01; ***P<0.001. Data is representative of duplicate independent experiments. (K) Counts of mitochondrial morphology were performed on 60 cells from duplicate experiments in control or PINK1 overexpressing cell lines. Differences were analyzed by two-way ANOVA using morphology and cell line/treatment as factors and *P* values for cell groups are given above each graph.

Next, we addressed what would happen if levels of Drp1 were increased in PINK1 deficient cells. We transiently expressed YFP-Drp1 in the same cell lines and then imaged mitochondria to detect YFP, using non-transfected (YFP negative) cells in the same cultures as controls (Fig. 6A–D). In both control and PINK1 shRNA lines, YFP-Drp1 was recruited to foci on the surface of mitochondria as described previously [40]–[42]. This punctate staining pattern was detected with both N- and C- terminal YFP fusions of Drp1, but not with YFP alone (data not shown) and represent fission competent foci of active Drp1. Twenty-four hours after transfection, PINK1 deficient lines showed more disrupted mitochondrial networks compared to control lines or untransfected cells (compare Figs. 6B and 6D). The amount of YFP-Drp1 recruited into mitochondrial fractions was similar in control and PINK1 deficient lines by immunoblot (Fig. 6E). Furthermore, we also noted that increased expression of YFP-Drp1 resulted in an accumulation of both transfected and endogenous Drp1 protein in mitochondria, again presumably due to the formation of fission-competent Drp1 oligomers at the mitochondrial surface, and again this effect is similar in both control and PINK1 shRNA cell lines.

Mitochondrial connectivity was quantitated by FRAP using an HA-tagged Drp1 construct to avoid interference of the YFP tag on FRAP. We confirmed that >90% of mito-YFP positive cells were HA-Drp1 positive (data not shown). As in the experiments above with mitotracker, YFP labeled mitochondria showed extensive fragmentation in PINK1 deficient cell lines when challenged by Drp1 overexpression (Fig. 6F). Drp1 overexpression decreased FRAP in both control shRNA and PINK1 shRNA cell lines, with the lowest mobile fraction values seen in the latter (Fig. 6G, 6H). Therefore loss of PINK1 and overexpression of Drp1 have additive effects. FRAP experiments also showed that overexpression of PINK1 limits Drp1-mediated mitochondrial fragmentation (Fig. 6J). The sensitivity of PINK1 deficient cells (Fig. 6I) and resistance of PINK1 overexpressing cells (Fig. 6K) to Drp1-mediated fragmentation was confirmed using counts of mitochondrial morphology. We also overexpressed the mitochondrial fusion proteins Optic Atrophy 1 (OPA1) and Mitofusin 2 (Mfn2) and found that either could recover mitochondrial connectivity in PINK1 knockdown cells (Supplementary Fig. S4). Overall, these results suggest that PINK1 deficient mitochondria are more sensitive to mitochondrial fission.

### Drp1 phosphorylation and activity are altered in the absence of PINK1 due to calcineurin activation

**Figure 7:**
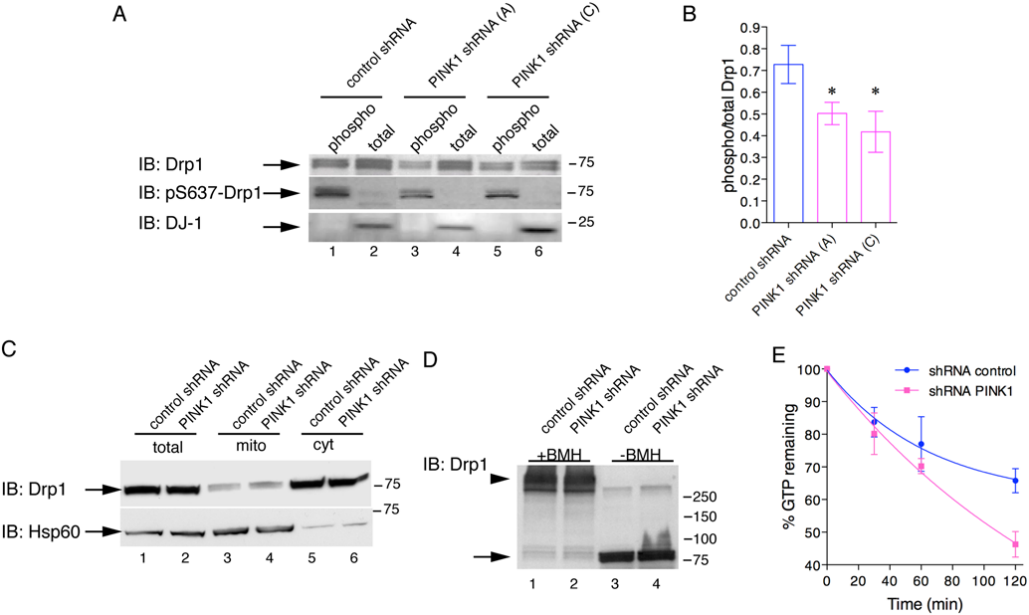
Dephosphorylation and increased GTPase activity of Drp1 in PINK1 deficient cells. (A) Lysates from control shRNA lines or either of two PINK1 shRNA lines were separated by phospho-enrichment compared to total lysates and blotted for the proteins indicated on the left of the blots. Drp1 was in both phospho-enriched and total fractions, but less phospho-Drp1 was seen in the PINK1 deficient cells. We used phospho-Drp1 to confirm the phosphopurification was efficient and DJ-1 served as a negative control. (B) Quantification of Drp1 as a ratio of the phospho-enriched and total fractions shows that there was an ∼30% decrease in phospho-Drp1 (*n* = 3) that is consistent between both shRNA sequences (*, *P*<0.05 by ANOVA). (C) Recruitment of endogenous Drp1 to mitochondrial fractions (mito) is similar in control and PINK1 shRNA cell lines (total: whole cell lysates, cyt: cytosolic fractions). Data are representative of duplicate experiments and molecular weight markers on the right of the blots are in kilodaltons. (D) Oligomerization status of Drp1 is not affected by PINK1 shRNA compared to control lines. Lysates were crosslinked with BMH or not treated to show equivalent loading of Drp1. Arrowhead shows Drp1 oligomers, arrow shows monomeric Drp1. Molecular weight markers are in kilodaltons. (E) GTPase activity of Drp1 immunopurified from control or PINK1 shRNA cell lines was followed over time and expressed as percentage of GTP converted to GDP. The difference between the lines was significant (*P*<0.01 by two-way ANOVA). Each point is the average of 3 replicates and is representative of two independent experiments.

Next, we wanted to know if Drp1 activity was modified in PINK1 deficient cells. As Drp1 activity can be affected by serine phosphorylation [40]–[43], we examined the phosphorylation status of Drp1 in PINK1 deficient cells by enriching for phosphoproteins and blotting for endogenous Drp1. We confirmed the phospho-enrichment by blotting for p-S637 Drp1 and used DJ-1, which is not basally phosphorylated [44], as a negative control (Fig. 7A). Drp1 showed a loss of phosphorylation in the absence of PINK1 (Fig. 7A). Quantification across multiple experiments showed an ∼30% loss of endogenous Drp1 phosphorylation in PINK1 deficient cells (Fig. 7B). Phosphorylated amounts of OPA1, Mfn1 and Mfn2 and HtrA2 were also examined, but no differences between cell lines were found (Supplementary Fig. S5). Subcellular fractionation of control versus PINK1 shRNA cells were analyzed by western blotting and show mitochondrial recruitment of endogenous Drp1 is not influenced by the loss of PINK1 (Fig. 7C). BMH-crosslinking experiments did not show any alterations in Drp1 oligomerization in PINK1 deficient cells (Fig. 7D). Using *in vitro* GTPase assays, we found increased GTPase activity of Drp1 in cells expressing PINK1 shRNA (Fig. 7E; *P*<0.01 for cell lines), which may be related to the decreased phosphorylation seen in the same lines.

**Figure 8:**
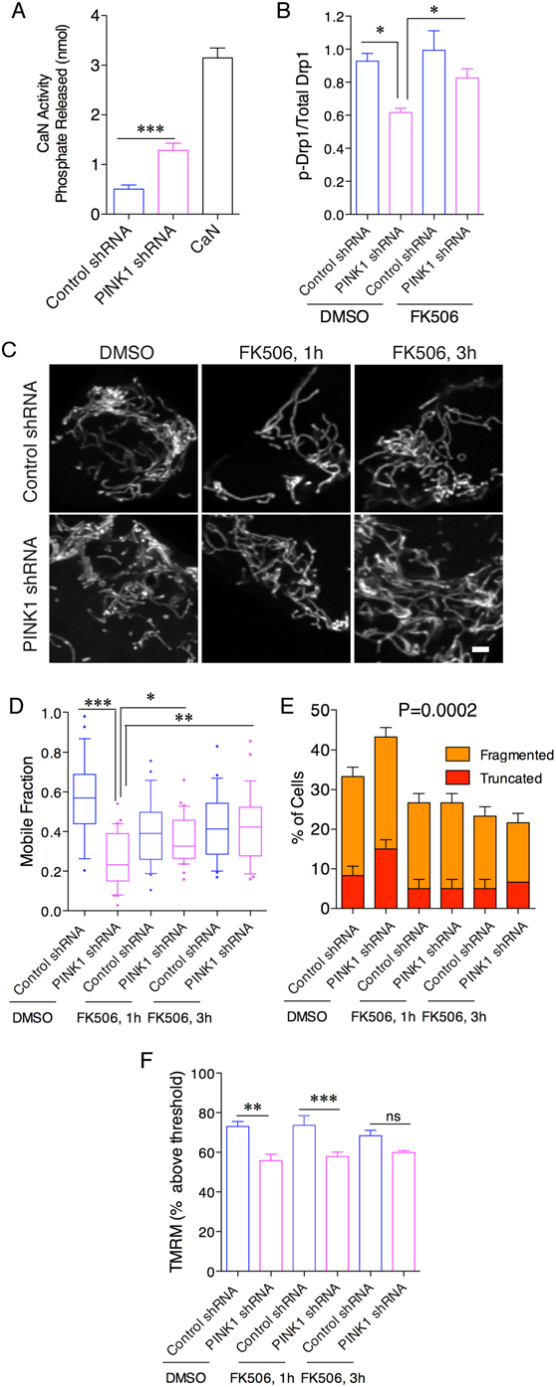
Calcineurin-mediated dephosphorylation of Drp1 contributes to mitochondrial phenotypes related to loss of PINK1. (A) Calcineurin enzyme activity was measured in postnuclear supernatants of extracts from control and PINK1 shRNA lines. Recombinant calcineurin (CaN) was used as a positive control. Activity is expressed as the release of Pi from a calcineurin substrate peptide, corrected for activity seen in the presence of EDTA to chelate calcium (***, *P*<0.001.) (B) Cell extracts from PINK1 shRNA lines, treated either with DMSO as a vehicle or 1 µM FK506 for 1 h and enrichment of Drp1 in the phosphorylated fraction was measured as in figure 6B. Quantification (*n* = 6) confirmed a lower relative amount of phospho-Drp1 in PINK1 shRNA cells compared to controls and a significant increase comparing PINK1 shRNA cells treated with DMSO to those treated with FK506 (*, *P*<0.05 by ANOVA). All other comparisons were not significant (*P*>0.05). (C) Mitochondrial morphology in control shRNA and PINK1 shRNA cells was assessed by mito-YFP expression. Cells were treated with vehicle only or 1 µM FK506 for 1 h or 3 h. Increasing length of FK506 treatment improves mitochondrial connectedness, especially in the PINK1 shRNA line. Scale bar is 2 µm and applies to all panels. (D) Mobile fraction values were estimated from FRAP curves either without treatment or after 1 h or 3 h treatment with 1 µM FK506. Each box is the average of 60 measurements from cells across duplicate experiments. *, *P*<0.05; **, *P*<0.01, ***, *P*<0.001 by one-way ANOVA with Student-Newman Kuells' *post-hoc* test. For clarity, non-significant differences (*P*>0.05) are not indicated. (E) Counts of mitochondrial morphology as in figure 2 were performed on n = 60 cells from duplicate experiments in control shRNA or PINK1 shRNA cell lines after treatment with FK506 for 1 or 3 hours. Differences were analyzed by two-way ANOVA using morphology and cell line/treatment as factors and *P* values for cell groups are given above each graph. (F) Mitochondrial membrane potential was estimated in PINK1 knockdown cells using TMRM staining and FACS analysis with and without 1 or 3 h treatment with 1 µM FK506. There is a significant (*, *P*<0.05; ***, *P*<0.001, *n* = 6 independent experiments) difference between the lines under basal conditions or after 1 h treatment by one-way ANOVA with Student-Newman Kuells' *post-hoc* test, although this is not significant (ns) at 3 h.

Calcineurin (CaN) increases Drp1 activity via its phosphatase activity [41]. Neither calcineurin catalytic α subunit, regulatory calcineurin-β subunit or calmodulin protein levels were altered between PINK1 deficient cells and controls (data not shown). However, cellular CaN activity was significantly increased in PINK1 deficient cells (Fig. 8A; *P*<0.001 by ANOVA, *n* = 5). To address whether CaN activity influences Drp1, we treated cells with 1 µM of the CaN inhibitor FK506 for 1 hour and separated phosphorylated and total proteins and immunoblotted for Drp1 as in figure 7B. Quantification of multiple experiments confirmed the decreased ratio of phosphorylated to total Drp1 in PINK1 deficient cells compared to control cells and showed that this measure of Drp1 phosphorylation was increased to a level similar to controls after FK506 treatment (*P*<0.05 by ANOVA, *n* = 6; Fig. 8B).

We also treated cells with 1 µM FK506 for 1–3 hours and imaged mitochondria using mito-YFP (Fig. 8C). Quantitatively, the lower FRAP signal in PINK1 deficient mitochondria was increased by FK506 treatment (Fig. 8D) such that by 3 h the difference between control and PINK1 shRNA lines was not significant. The effect of FK506 in PINK1 shRNA lines was significant at 1 h (*P*<0.05) and 3 h (*P*<0.001 by ANOVA, *n* = 60 cells measured over two experiments). Cell counts were also performed and confirmed that FK506 decreases the number of truncated or fragmented mitochondria (*P* = 0.0002; Fig. 8E). We investigated whether the rescue in connectivity would affect mitochondrial membrane potential and therefore we treated control and PINK1 shRNA lines with FK506 1 µM for 1 and 3 h and measured TMRM. There was no increase in TMRM intensity in PINK1 deficient cells (Fig. 8F), suggesting that that calcineurin-mediated dephosphorylation of Drp1 does not influence these events.

## Discussion

Mitochondrial dysfunction has been linked to neuronal cell loss in PD and it seems likely that the genes associated with recessive parkinsonism help maintain mitochondrial function under stressful conditions. Here, we first confirm previous reports suggesting that PINK1 is protective against mitochondrial toxins [14], [30], [31], [33] and that PINK1 deficiency results in lower Δψ_m_, [12], [14]. Furthermore, we demonstrate that in a mammalian system, mitochondrial fragmentation is promoted by PINK1 silencing through a mechanism that involves calcineurin-mediated dephosphorylation of the GTPase, Drp1.

The dynamic regulation of mitochondrial morphology is important in maintaining the health of the organelle and of the cell [25], [45], [46]. Mitochondria with low Δψ_m_ are preferentially degraded by autophagic turnover [46]–[49] and recent data suggests that parkin [7] and PINK1 [15] can promote this process for compromised organelles. Parkin can rescue loss of PINK1 phenotypes [8]–[10], [12] and it is possible that both proteins form a linear pathway to remove damaged mitochondria. Our data would therefore be consistent with a model whereby lack of PINK1 or parkin results in the accumulation of mitochondria with lower Δψ_m_.

**Figure 9:**
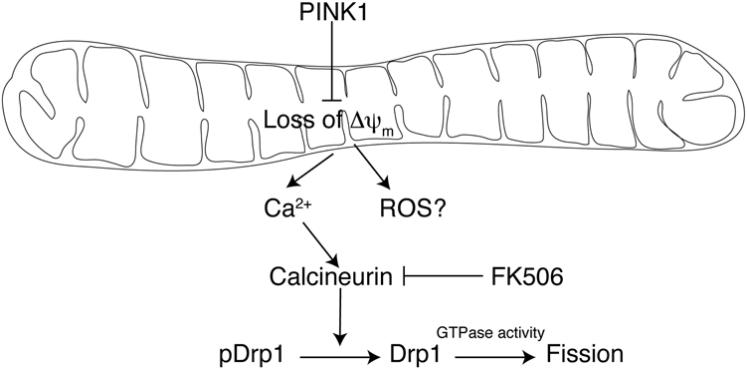
Proposed mechanism of altered mitochondrial morphology induced by loss of PINK1.

Many aspects of mitochondrial function are dependent on Δψ_m_. This includes the homeostatic control of cytosolic Ca^2+^ by mitochondria [50], [51], a process that involves interactions with the endoplasmic reticulum [52], and requires the mitochondrial dynamics protein Mfn2 [53]. Recent results have suggested that cells overexpressing mutant forms of PINK1, which may have a dominant negative effect, have a number of alterations in mitochondria that are dependent on uptake of Ca^2+^ into the organelle [54]. Moreover, cells lacking PINK1 have increased calcium efflux from mitochondria via the mitochondrial Na^+^/Ca^2+^ exchanger [16]. Our data shows that PINK1 deficient cells have higher activity of the Ca^2+^-dependent phosphatase calcineurin, which is known to dephosphorylate Drp1 and influences susceptibility to cell death [41], [55]. Drp1-dependent mitochondrial fragmentation is influenced by changes in calcium levels [56] and by phosphorylation at Ser637 [40]. We propose that calcineurin, which we speculate might be activated by increased local Ca^2+^ near compromised mitochondria, will dephosphorylate Drp1 and thus lead to mitochondrial scission as a secondary event. This hypothesis would be consistent with our data that the calcineurin inhibitor FK-506 rescues FRAP defects but has only modest effects on Δψ_m_ (Figure 9), although this will need to be confirmed in further studies.

It should be noted that this proposal does not necessarily mean that PINK1 controls Δψ_m_ directly as it may simply be the case that PINK1 deficient cells accumulate a population of mitochondria with low Δψ_m_ due to decreased mitophagy. As well as derangements of Δψ_m_ and calcium, several recent results indicate that increased reactive oxygen species contribute to mitochondrial dysfunction in PINK1 [15] and parkin [13] deficiency models. Therefore, the overall model of PINK1 influence on mitochondria, as we have outlined in figure 9, probably includes several inter-related events.

Our data differ somewhat from *Drosophila* models, where increased Drp1 dosage suppresses effects of PINK1 deficiency, leading to the proposal that PINK1 promotes fission [22], [23], [36], [57]. In contrast, measuring mitochondrial connectivity in mammalian cells, we find that decreasing Drp1 had a suppressive effect and, measuring fusion, we observed slower loss of pA-GFP in PINK1 deficient cells. One possible explanation for the apparent difference between our results, which are consistent with some other results in human neuroblastoma cell lines [12], [15], and those in *Drosophila* models is that the effects of PINK1 on mitochondrial morphology are indirect. It has been suggested that excess fusion can buffer accumulated damage in parkin-deficient fly muscle [58] and is a response to the damage rather than a cause. A similar interpretation of the various data from *Drosophila*, namely that PINK1 is not a core component of the fusion/fission machinery, has been proposed [36]. If the primary event is accumulation of compromised mitochondria, then the effects on morphology are secondary and may vary depending on tissue type and stage. In the case of the mammalian cells used here, the output is a loss of connectivity due to secondary effects on Drp1.

Using electron microscopy, we see an approximate doubling in the number of cells with mitochondria that contained swollen cristae. Similar results are also seen in other cell lines [54], and in one recent study using EM an association of autophagic vacuoles with mitochondria with fewer cristae per unit length was noted [15]. Mitochondrial swelling is very prominent in the flight muscles of PINK1 deficient *Drosophila* [8]. Whether this is related to fission or fusion is unclear, as swelling might be independent of either process, but this does indicate that some aspects of mitochondrial dysfunction are conserved in different species when PINK1 is removed. It is also important to note that parkin can rescue PINK1 deficiency in both *Drosophila* [8], [9] and in mammalian cell lines [12], [15]. However, there are clear species and/or tissue differences with respect to the effects of PINK1, as experiments in mouse brain have not revealed any obvious changes in mitochondrial morphology in the absence of exogenous stressors [18].

It is possible that the effect of PINK1 is to influence mitochondrial fusion, in which case the effects of Drp1 would be antagonistic but indirect. In an attempt to address this directly, we used photoactivatable mitochondrially directed GFP and measured the rate of loss of pA-GFP, which should correlate to decreased rates of fusion. However, this data is not straightforward to interpret if PINK1 plays a role, for example, in mitochondrial transport. We have recently described that PINK1, probably on the cytoplasmic face of mitochondria [59], interacts with Milton and Miro [60], two proteins involved in mitochondrial transport. Therefore, the decreased pA-GFP may relate to either a decreased mitochondrial transport rate, decreased mitochondrial fusion or both. Further experiments, perhaps using dual labeled mitochondria, are required to resolve this issue. However, what is relatively clear is that loss of PINK1 does not result in increased mitochondrial fusion [36], at least in this model system.

An important point that arises from comparing different studies on mitochondrial function under circumstances where PINK1 activity has been depleted is that there are many different assays to address function and morphology of this complex organelle. In the current study, we principally used FRAP and pA-GFP as ways to interrogate mitochondria in living cells, which we chose so that we could avoid the influence of fixation or other processing steps. However, each of these methods has important advantages and disadvantages that should be considered in terms of the physical nature of mitochondria. For example, in some experiments we measured mitochondrial length and found that, as expected, lowering Drp1 levels increases mitochondrial length. However, at the light microscope level it is difficult to separate two nearby and juxtaposed mitochondria from one convoluted organelle, given their morphology. Therefore, we used FRAP to estimate total connected matrix volume, which is related to morphology and in our hands this is sensitive to relatively subtle changes. However, one of the limitations of this technique is the limited dynamic range and the wide variety of signal across different cells in a population, which necessitates measuring relatively large numbers of cells and validation by adding known fusion or fission molecules. As discussed above, the pA-GFP measures, which in principle should directly measure fusion, might also be influenced by mitochondrial transport. Therefore, our results need to be confirmed by different techniques performed in similar models. In this context, Dagda et al used a different estimate of mitochondrial connectivity, namely perimeter/area ratio measurements from fluorescently labeled mitochondria and found that PINK1 decreases connectivity [15].

Overall, our data support a role of PINK1 in limiting the damaging effects of loss of mitochondrial function. These results will allow us to investigate pathways relevant to dopaminergic cell death in further detail.

## Materials and Methods

### Cell lines and lentiviral vectors

Human BE(2)-M17 (ATCC designation CRL-2267) human neuroblastoma cells [61]–[63] were used as a model for dopaminergic neuron-like cells as they express dopamine synthesis enzymes [64] and have measurable levels of dopamine (DW Miller, unpublished observations). Cells were maintained in Optimem I media (Invitrogen) with 10% FBS. Cell lines were selected with 5 µg ml^−1^ blasticidin (Invitrogen) for several weeks until non-transduced cells were dead. We used cells within fifteen passages of transduction. Both the overexpression vectors and the shRNA vectors encode blasticidin resistance cassettes, so the selection and maintenance are the same for both sets of lines. Where parental cells (here referred to as M17) were used, we did not include blasticidin in the growth media and all experiments using live cell imaging were performed similarly in the absence of blasticidin.

The cDNA for human PINK1 and mutants described previously [29] were subcloned into pLenti6 (Invitrogen) and packaged into viral particles according to the manufacturer's instructions. Cells were transduced at a multiplicity of infection (MOI) of approximately 1 and stable clones were established by selection with Blasticidin (Invitrogen). To knockdown endogenous PINK1, we made two separate shRNA constructs (target sequences 5′-GCTGGAGGAGTATCTGATAGG, starting at nucleotide 550 of human PINK1 and 5′-GGGAGCCATCGCCTATGAAAT, starting at nucleotide 1411) and a control shRNA (5′- CCTAGACGCGATAGTATGGAC). These sequences were cloned into pLenti6, packaged and used to transduce M17 cells as above. Knockdown was confirmed using quantitative reverse transcriptase-polymerase chain reaction (qRT-PCR) methods described previously [29].

### Western blotting

General methods for western blotting for PINK1 expression using total SDS cell lysates have been described previously [29]. For mitochondrial and cytosolic separations, we used the mitochondrial isolation kit for cultured cells (Pierce), according to the manufacturer's instructions. Phosphoprotein enrichment was performed and validated as described previously [65]. The following antibodies were used at given dilutions; monoclonal anti-V5 (Invitrogen), 1∶500; monoclonal anti-βactin (Sigma), 1∶5000; monoclonal anti-Drp1 (BD Translabs), 1∶1000; monoclonal anti-Opa1 (BD Translabs), 1∶500; polyclonal anti-Omi/HtrA2 (Cell Signaling), 1∶1000; polyclonal anti-Fis1 (Biovision) 1∶1000; monoclonal anti-Hsp60 (Stressgen), 1∶1000; monoclonal anti-DJ1 (Stressgen), 1∶1000. Mfn1 and Mfn2 antibodies were a generous gift from Dr. Richard Youle. Phospho-S637 Drp1 antibody was kindly provided by Dr. Craig Blackstone.

### Cell viability

Cell viability was measured using Annexin V and PI staining to label apoptotic and necrotic cells respectively using the Vybrant Apoptosis Assay Kit (Molecular Probes). Cultured cells were harvested from 24 well plates by 37°C Tryple (Invitrogen) treatment and then suspended with PBS and we pooled floating cells with harvested cells to give an estimate of the total cell viability across the total population of cells in each well. The cells were washed by centrifugation at 1500×*g* at 4°C for 5 min and pellets were resuspended in 100 µl 1× annexin-binding buffer mixed with annexin V-FITC and PI for 15 min at room temperature in the dark. 200 µl 1× annexin-binding buffer was then added to the stained cells, followed by mixing and keeping the tubes on ice. The stained cells were analyzed immediately by flow cytometry. As a positive control, cells were exposed to 20 mM H_2_O_2_ for 10 minutes at 37°C, which evokes rapid cell death. Flow cytometry data were plotted as a function of fluorescence intensity FL-1 (green) versus FL-3 (red) fluorescence. We used annexin V-FITC (emission 518 nm) versus PI (emission 617 nm) to identify populations of viable cells (annexin V^−^PI^−^), early apoptotic cells (annexin V^+^PI^−^), necrotic cells (annexin V^−^PI^+^) and late necrotic cells (annexin V^+^PI^+^). Flow cytometry analysis was performed with a FACS Calibur (Becton Dickinson, San Jose, CA) equipped with a 488-nm argon laser and a 635-nm red diode laser. Experiment data were analyzed in CellQuest software (Becton Dickinson) using 5–10,000 events per sample. Triplicate experiments were performed. MTT assays were performed as described previously [3].

### Analysis of Mitochondrial Membrane Potential (Δψ_m_) by Flow Cytometry

Cells were harvested, washed once with PBS and stained with 100 nM tetramethyl rhodamine methyl ester (TMRM) for 15 minutes at 37°C, then washed once with PBS and kept at 4°C during measurements. Cytofluorimetric analysis was performed using a FACScan flow cytometer (Becton-Dickinson, San Jose, CA) equipped with a 488 nm argon laser. TMRM signal was analyzed in the FL2 channel and the data were acquired on a logarithmic scale. Non-cellular debris and dead cells were gated out based on light-scattering properties in the Side- and forward-scatter parameters, and 10–20,000 events from live cells were collected for each analysis. Data were analyzed using the CellQuest software (BD) and exposure to 10–100 µM CCCP (3-chlorophenylhydrazone) for 10 minutes was used to set a threshold of fluorescence intensity for those cells with intact Δψ_m_ (Supplementary figure S1). Results are expressed as % of all cells with TMRM fluorescence greater than the threshold set by CCCP.

### Confocal Microscopy

Cells were seeded on 22-mm coverslips coated with poly-L-lysine. Mitochondria were stained with 200 nM Mitotracker CMXRos (Molecular Probes, Inc.) for 30 minutes at 37°C, fixed with 4% paraformaldehyde, washed and mounted with ProLong Gold Antifade reagent (Molecular Probes, Inc.). Counts for mitochondrial morphologies were performed by counting 45–130 Mitotracker stained cells in each of three independent experiments. The observer was blind to transfection/treatment status. In some experiments, we additionally measured mitochondrial length from images of cells transfected with mitochondrially-directed YFP using the imaging software ImageJ. We thresholded images based on background fluorescence and measured total length of objects above background.

### Transmission electron microscopy

Cells were fixed in 2% glutaraldehyde in 0.1 M sodium cacodylate buffer containing 0.1 M sucrose and 3 mM CaCl_2_, pH 7.4 at room temperature for 30 min then overnight at 4°C. After fixation, cells were rinsed in 0.15 M sodium cacodylate buffer containing 3 mM CaCl_2_, pH 7.4 and centrifuged. Pellets were then postfixed in 2% osmium tetroxide in 0.07 M sodiumcacodylate buffer containing 1.5 mM CaCl_2_, pH 7.4 at 4°C for 2 hour, dehydrated in ethanol followed by acetone and embedded in LX-112 (Ladd, Burlington, Vermont, USA). Sections were contrasted with uranyl acetate followed by lead citrate and examined in a Tecnai 10 transmission electron microscope (Fei, The Netherlands) at 80 kV. Digital images were taken by using a MegaView III digital camera (Soft Imaging System, GmbH, Münster, Germany)

### Measurements of mitochondrial connectivity

FRAP was performed as previously described [66], [67]. Circular ROIs, 2.5 µm in diameter, were imaged over a perinuclear region of the cytoplasm that contained highly interconnected mitochondria, using a 100× Plan-Apochromat 1.4/Oil DIC objective lens (Carl Zeiss) before and after photobleach with 4 iterations of 514-nm laser set to 100% power. Scans were taken in 0.25 second intervals, for a total of 40 images and the fluorescence intensity in imaged ROIs was digitized with LSM 510 software (Zeiss MicroImaging). Curves were corrected for both non-specific photobleaching (NSPB) that occurred during imaging and background, and normalized to the first image in the series. Each FRAP curve represents the average of ≥30 measurements representative of results obtained in 2–3 separate experiments. Mobile Fractions [68] were calculated as follows: Mobile Fraction = [(FRAP_t_−Background)/FRAP_i_][(NSPB_i_−Background)/NSPB_t_].

### Photoactivation using live cell imaging with confocal microscopy

Assays for mitochondrial fusion rates were performed using modifications of techniques described previously [66]. Briefly, cells were transfected as above with photoactivatable mitochondrially targeted GFP (mito-paGFP). Cells were imaged using a 63× plan-apochromat 1.4/oil DIC objective lens (Zeiss). After collecting baseline fluorescence images, two circular, 15 µm, regions were photoactivated in each cell using a 413-nm laser set to 50% power output and 100% excitation. Fluorescence images were then taken of the same cells (n = 9–10 per line with N = 3 experiments performed) at 15 minute intervals over one hour using a 488 nm laser with 5% excitation and 70% laser power, with a GFP filter for emission. Fusion of mitochondria was estimated from the loss of (photoactivated) mito-paGFP fluorescence, averaged across both regions of interest and normalized to the initial measurement, which was set at 100% (MetaMorph software, Molecular Devices).

### BMH Crosslinking of Drp1

Prior to harvest, cells were incubated with BMH crosslinker (20 µM, 30 min) followed by two rinses with DTT (20 mM) to quench the crosslinking reaction. Cell lysates were separated via gel electrophoresis and protein multimers were detected on immunoblots using monoclonal antibodies for Drp1, Bax -clone 3 (BD Transduction Labs) and DJ-1 (Stressgen). M17 cells treated with staurosporine (400 nM, 6 hr) served as a positive control for apoptosis-related multimerization of Drp1 and Bax.

### GTPase Assay

Cells were lysed and centrifuged to remove insoluble material. Supernatants were immunoprecipitated with 4 µg anti-Drp1 antibody (BD Translabs) overnight with rocking at 4°C. Lysates were then incubated with protein G sepharose beads (Amersham) for 2 hrs at 4°C. Beads were washed 5 times with PBS supplemented with 300 mM NaCl and 1% Triton X-100, once in assay buffer (20 mM HEPES pH 7.2, 2 mM MgCl2, 1 mM DTT, 0.005% BSA), re-suspended in 40 µl of the same buffer and α^32^P-GTP (5 µ Ci; GE healthcare) was added to each reaction. Samples were incubated at room temperature with vigorous shaking and 1 µl aliquots removed at time points from 0–120 minutes and spotted onto TLC plates (Sigma). Samples were then subjected to rising thin layer chromatography under 1 M formic acid, 1.2 M LiCl for two hours. Plates were dried for 5 min and radioactive bands were detected by autoradiography using a phosphoscreen. [^32^P]-GDP and [^32^P]-GTP spots were identified using a Storm860 PhosphorImager with ImageQuant software (GE Healthcare). GTPase activity was expressed as loss of GTP at each time point. All data points represent the average of at least three independent experiments.

### Calcineurin activity assay

Post-nuclear cell extracts were desalted by gel filtration to remove excess phosphate and nucleotides. Samples were used in the cellular calcineurin (PP2B) phosphatase assay according to the manufacturer's instructions (Biomol). The detection of free-phosphate released is based on malachite green assay using human recombinant calcineurin as a positive control. Following background subtraction, OD620nm data was converted into the amount of phosphate released using standard curve line-fit data, where the amount of phosphate released = (OD620nm−Yint)/slope. To determine the contribution of calcineurin, activity from samples treated with EGTA were subtracted from the total phosphatase activity for each sample.

### Statistical analyses

Where a single output variable was compared for multiple groups, including all mobile fraction values in FRAP assays, we used one-way ANOVA to estimate *P* values for all groups. Where the overall *P* value was <0.05, Student-Newman-Kuell's *post hoc* tests were then used to perform multiple comparisons for each group. Where an experiment had multiple outcome groups, for example when cells were counted for viable/apoptotic/necrotic or mitochondria were counted for intact/truncated/fragmented, we used two-way ANOVA with morphology as one factor and all cell/lines treatments as another, reporting the overall *P* value for the groups. We also used two-way ANOVA for output measures that were time dependent, as in the photoactivation assays, using time and cell lines as factors, and again report overall *P* values.

## Supporting Information

Figure S1 Estimation of mitochondrial membrane potential using TMRM. Prior to FACS analysis, several groups of cells were treated with 1–100 µM CCCP to depolarize mitochondria (blue, red and black traces as indicated) with untreated cells used as a control group (green histogram). Cells were stained with TMRM, with an additional group analyzed without TMRM staining as indicated. Fluorescence intensity of TMRM was measured across 10,000 events and counts are shown on the y-axis. In the CCCP treated cells, there is a concentration-dependent shift towards lower intensity with a distinct inflection in the data, which was used to set the threshold. (B and C) are plotted from several similar experiments showing (B) the proportion of events where TMRM is greater than the threshold set for each experiment (this data is also in the main text as figure 1D) or (C) the mean TMRM fluorescence plotted using control shRNA cells measured in the same experiment as 100%. Both ways of expressing the data show a substantial loss of TMRM fluorescence across the cell population.
(1.16 MB TIF)

Figure S2 PINK1 is protective against mitochondrial toxins but does not protect against apoptotic cell death. (A–D) MTT assays were used to confirm FACS analyses (Figure 1) that PINK1 (pink lines, upwards triangles) protects against exposure to rotenone for 48 hours compared to cells expressing LacZ (blue squares), G309D PINK1 (green circles) or kinase dead PINK1 (black squares). The difference between cell lines was significant by two-way ANOVA (P<0.0001) as was the effect of rotenone (P<0.0001) and there was a significant (P<0.0001) interaction between the two parameters. A similar protective effect was seen for MPP+ (B; P<0.0001 for MPP+ concentration and P<0.0001 for cell line), but no protection was seen against the proteasome inhibitor MG132 (C) or the kinase inhibitor staurosporine (D). Each point represents the mean of n = 8 measurements, normalized to untreated cells in the same cell line. (E and F) Rotenone does not induce biochemical events typical of apoptosis. M17 cells were untreated (lanes 1,2), treated with 200 nM rotenone for 48 hours (lanes 3,4) or 100 nM staurosporine overnight (lanes 3,4). In E, cells were pretreated with the crosslinking agent BMH for 30 minutes prior to extraction and blotting for Bax. Arrow indicates monomeric Bax and arrowhead shows multimers of Bax typical of apoptotic cells seen after staurosporine treatment. (F) Immunoblot for PARP1 cleavage after staurosporine treatment-100 kDa PARP1 protein (arrow), 85 kDa fragment (arrowhead). Cells treated with toxic concentrations of rotenone do not show either Bax multimerization or PARP cleavage. Markers are in kilodaltons. For both blots, the vertical white between lanes 4 and 5 line shows where the blots were rearranged for clarity, but the images are from the same scan of the same blot and are thus comparable.
(1.54 MB TIF)

Figure S3 FRAP analyses in cell lines expressing alternate shRNA sequences. A similar analysis to that in figure 3E,F in the main text showing FRAP over time (A) and calculated mobile fraction of mito-YFP (B) in living cells. This is an independent control cell line (blue circles) and a different shRNA sequence (magenta squares) from those in figure 2. Open symbols are untreated cells, closed symbols are cells treated with 100 nM rotenone for 24 hours prior to imaging. Error bars indicate the SEM from 15 cells. Differences between untreated and treated cells were assessed from the summary data (B) using one-way ANOVA with Student-Newman Kuells' post-hoc test ** P<0.01; ***P<0.001 (n = 15 cells measured). (C) Parental M17, control shRNA and PINK1 shRNA cells were compared directly in FRAP experiments. Mobile fractions were plotted as in (B) and show that while there is no significant difference between M17 (black bars) and control shRNA (blue bars), the PINK1 deficient cells (magenta bars) are significantly different from either of the control lines using one-way ANOVA with Student-Newman Kuells' post-hoc test ** P<0.01; ***P<0.001 (n = 30 cells measured).
(0.45 MB TIF)

Figure S4 The fusion proteins Opa1 and Mfn2 rescue FRAP defects caused by loss of PINK1. (A) Control (upper panels) or PINK1 shRNA (lower panels) lines were imaged as in figure 3 with mito-YFP. Cells were either not transfected (UT, left panels) or transfected with the fusion proteins Opa1 (middle panels) or Mfn2 (right panels), which increased mitochondrial length. Scale bar is 2 µm, applies to all panels. (B, C) FRAP measurements were used to show that Opa1 improves connectivity of PINK1 shRNA cells (pink squares; open symbols are untransfected, closed symbols are with Opa1) but has only minor effects on the control shRNA cells (blue circles). Each time point is the average of 30 individual cells and is representative of duplicate experiments. Box plots in C summarize data from n = 30 cells (see figure 3 for explanation). *, P<0.05 for one-way ANOVA with Student-Newman Kuell's posthoc tests.
(1.28 MB TIF)

Figure S5 Analysis of additional proteins using phosphopurification in control and PINK1 shRNA cell lines To control for specificity in alterations of phospho-Drp1 in PINK1 knockdown cells, we examined the GTPases Opa1, Mfn1 and 2 and the protease Omi/HtrA2. Representative of triplicate independent experiments and purifications, no differences between cell lines were shown. Molecular weight markers are in kilodaltons.
(0.34 MB TIF)
