# Supplementary figures and images for: Mitochondrial Alterations in PINK1 Deficient Cells Are Influenced by Calcineurin-Dependent Dephosphorylation of Dynamin-Related Protein 1
Source: PLoS One. 2009 May 27;4(5):e5701. doi: 10.1371/journal.pone.0005701 (PMC2683574; doi:10.1371/journal.pone.0005701)

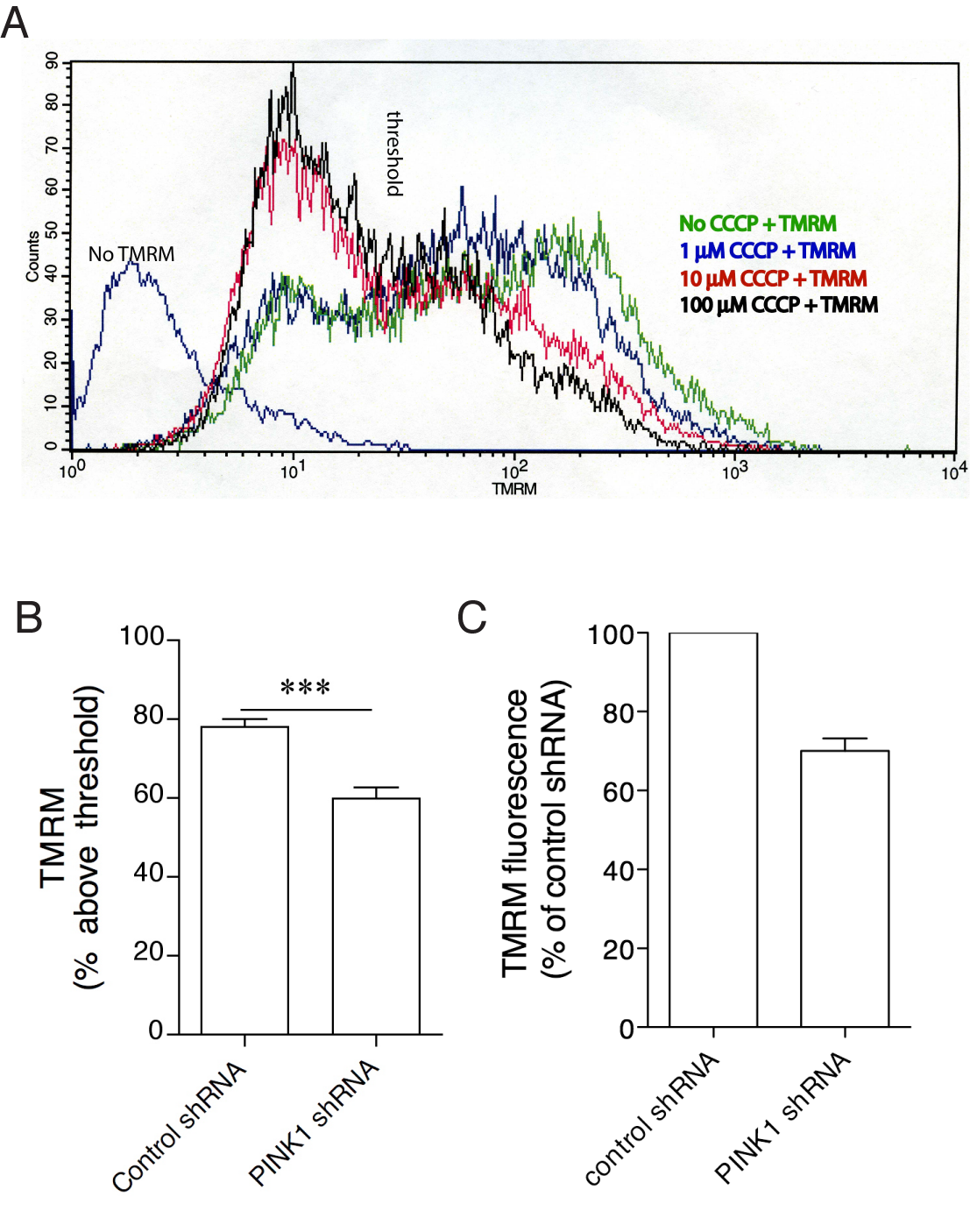

Supplement: Figure S1 — Estimation of mitochondrial membrane potential using TMRM. Prior to FACS analysis, several groups of cells were treated with 1–100 µM CCCP to depolarize mitochondria (blue, red and black traces as indicated) with untreated cells used as a control group (green histogram). Cells were stained with TMRM, with an additional group analyzed without TMRM staining as indicated. Fluorescence intensity of TMRM was measured across 10,000 events and counts are shown on the y-axis. In the CCCP treated cells, there is a concentration-dependent shift towards lower intensity with a distinct inflection in the data, which was used to set the threshold. (B and C) are plotted from several similar experiments showing (B) the proportion of events where TMRM is greater than the threshold set for each experiment (this data is also in the main text as figure 1D) or (C) the mean TMRM fluorescence plotted using control shRNA cells measured in the same experiment as 100%. Both ways of expressing the data show a substantial loss of TMRM fluorescence across the cell population. (1.16 MB TIF) [file pone.0005701.s001.tif]

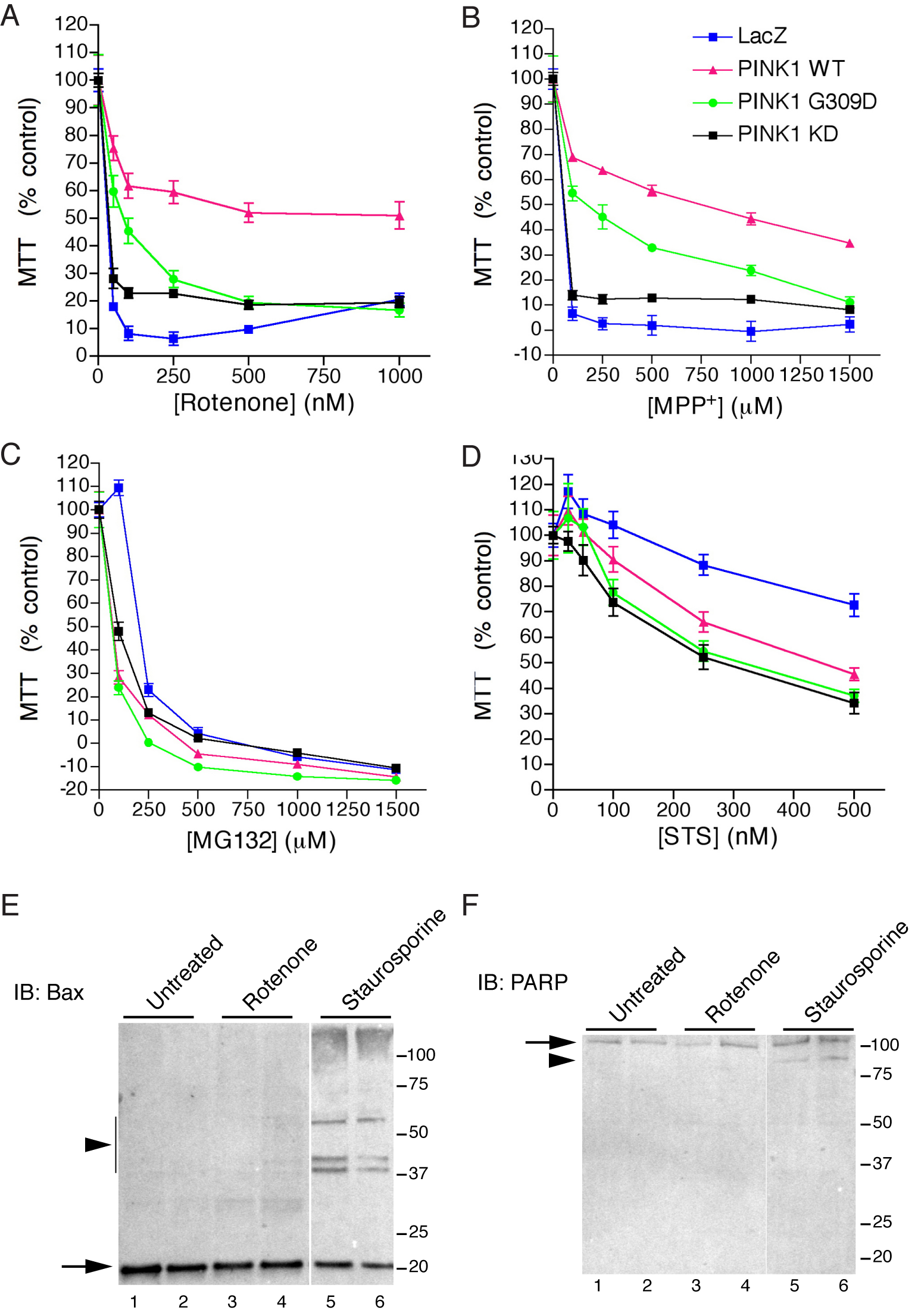

Supplement: Figure S2 — PINK1 is protective against mitochondrial toxins but does not protect against apoptotic cell death. (A–D) MTT assays were used to confirm FACS analyses (Figure 1) that PINK1 (pink lines, upwards triangles) protects against exposure to rotenone for 48 hours compared to cells expressing LacZ (blue squares), G309D PINK1 (green circles) or kinase dead PINK1 (black squares). The difference between cell lines was significant by two-way ANOVA (P<0.0001) as was the effect of rotenone (P<0.0001) and there was a significant (P<0.0001) interaction between the two parameters. A similar protective effect was seen for MPP+ (B; P<0.0001 for MPP+ concentration and P<0.0001 for cell line), but no protection was seen against the proteasome inhibitor MG132 (C) or the kinase inhibitor staurosporine (D). Each point represents the mean of n = 8 measurements, normalized to untreated cells in the same cell line. (E and F) Rotenone does not induce biochemical events typical of apoptosis. M17 cells were untreated (lanes 1,2), treated with 200 nM rotenone for 48 hours (lanes 3,4) or 100 nM staurosporine overnight (lanes 3,4). In E, cells were pretreated with the crosslinking agent BMH for 30 minutes prior to extraction and blotting for Bax. Arrow indicates monomeric Bax and arrowhead shows multimers of Bax typical of apoptotic cells seen after staurosporine treatment. (F) Immunoblot for PARP1 cleavage after staurosporine treatment-100 kDa PARP1 protein (arrow), 85 kDa fragment (arrowhead). Cells treated with toxic concentrations of rotenone do not show either Bax multimerization or PARP cleavage. Markers are in kilodaltons. For both blots, the vertical white between lanes 4 and 5 line shows where the blots were rearranged for clarity, but the images are from the same scan of the same blot and are thus comparable. (1.54 MB TIF) [file pone.0005701.s002.tif]

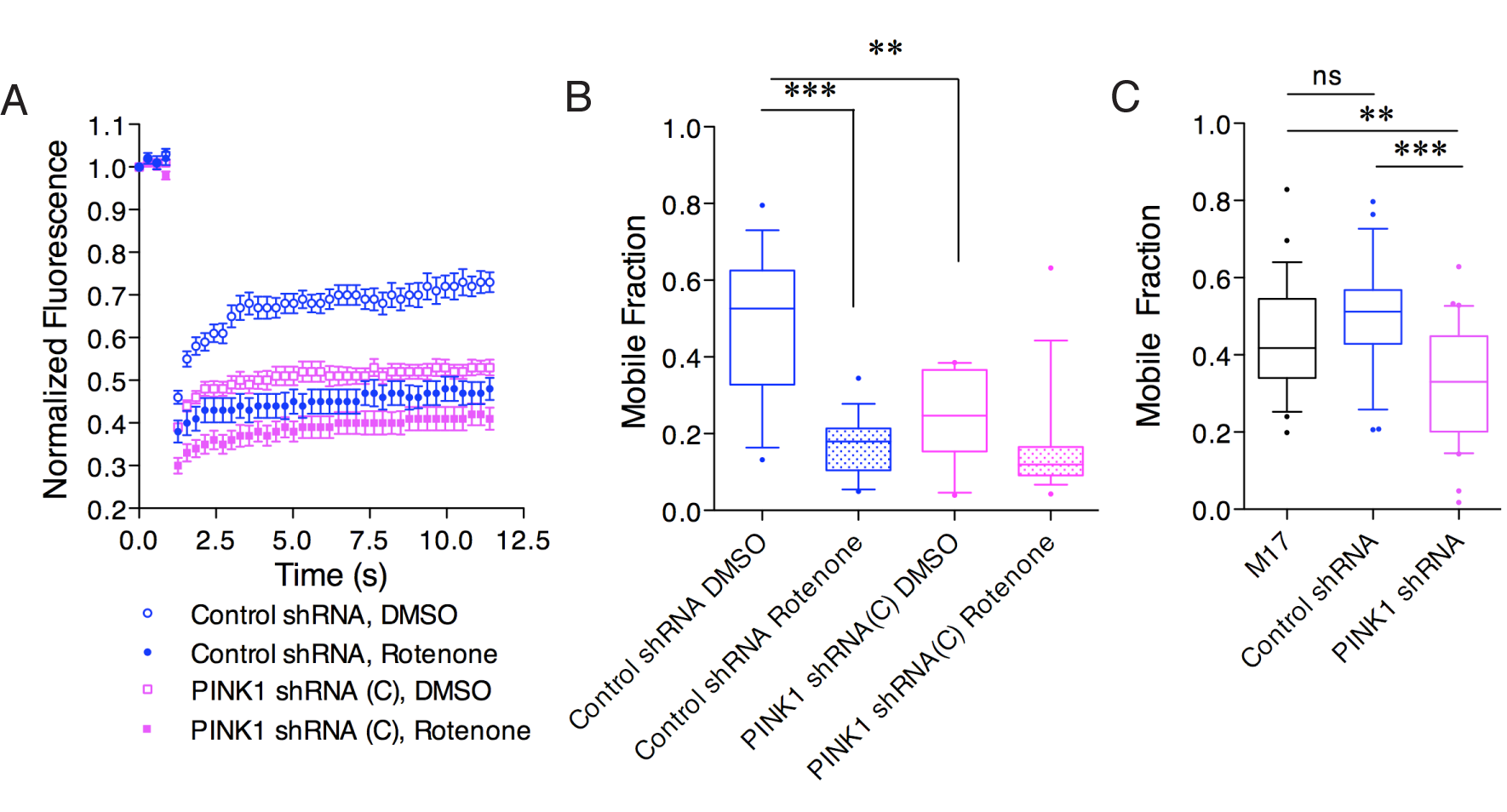

Supplement: Figure S3 — FRAP analyses in cell lines expressing alternate shRNA sequences. A similar analysis to that in figure 3E,F in the main text showing FRAP over time (A) and calculated mobile fraction of mito-YFP (B) in living cells. This is an independent control cell line (blue circles) and a different shRNA sequence (magenta squares) from those in figure 2. Open symbols are untreated cells, closed symbols are cells treated with 100 nM rotenone for 24 hours prior to imaging. Error bars indicate the SEM from 15 cells. Differences between untreated and treated cells were assessed from the summary data (B) using one-way ANOVA with Student-Newman Kuells' post-hoc test ** P<0.01; ***P<0.001 (n = 15 cells measured). (C) Parental M17, control shRNA and PINK1 shRNA cells were compared directly in FRAP experiments. Mobile fractions were plotted as in (B) and show that while there is no significant difference between M17 (black bars) and control shRNA (blue bars), the PINK1 deficient cells (magenta bars) are significantly different from either of the control lines using one-way ANOVA with Student-Newman Kuells' post-hoc test ** P<0.01; ***P<0.001 (n = 30 cells measured). (0.45 MB TIF) [file pone.0005701.s003.tif]

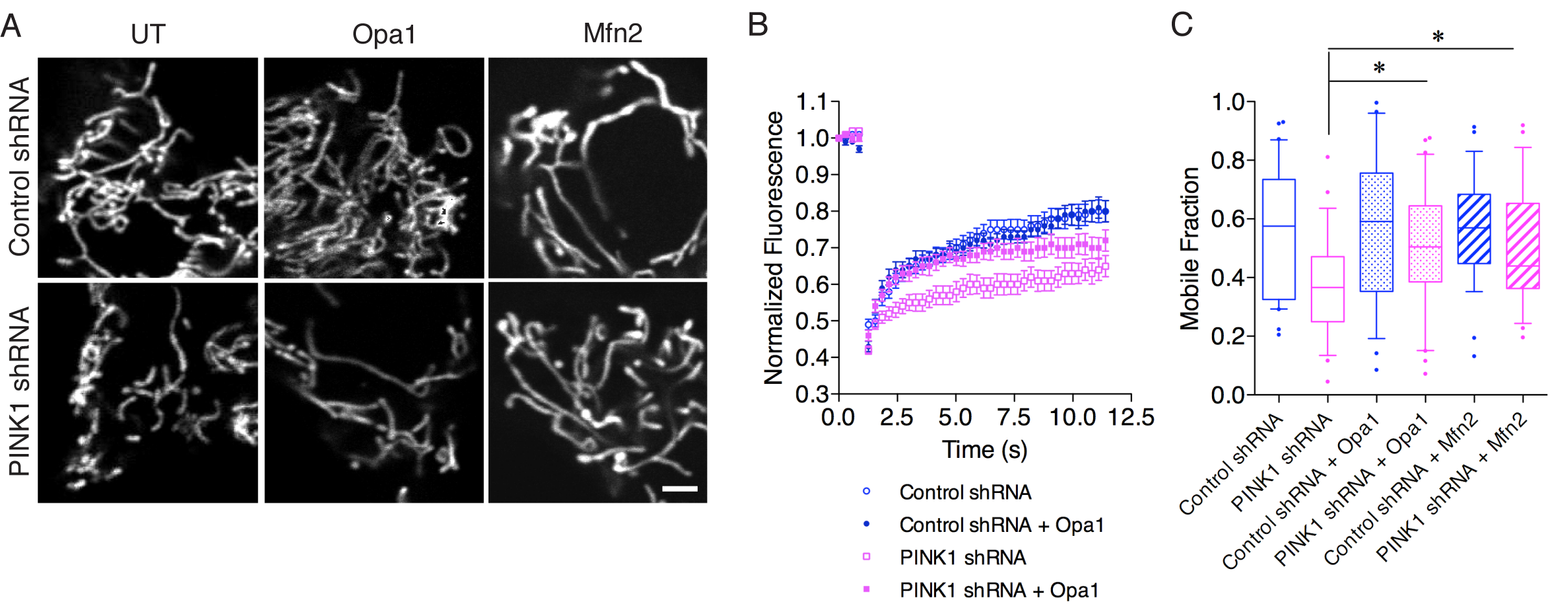

Supplement: Figure S4 — The fusion proteins Opa1 and Mfn2 rescue FRAP defects caused by loss of PINK1. (A) Control (upper panels) or PINK1 shRNA (lower panels) lines were imaged as in figure 3 with mito-YFP. Cells were either not transfected (UT, left panels) or transfected with the fusion proteins Opa1 (middle panels) or Mfn2 (right panels), which increased mitochondrial length. Scale bar is 2 µm, applies to all panels. (B, C) FRAP measurements were used to show that Opa1 improves connectivity of PINK1 shRNA cells (pink squares; open symbols are untransfected, closed symbols are with Opa1) but has only minor effects on the control shRNA cells (blue circles). Each time point is the average of 30 individual cells and is representative of duplicate experiments. Box plots in C summarize data from n = 30 cells (see figure 3 for explanation). *, P<0.05 for one-way ANOVA with Student-Newman Kuell's posthoc tests. (1.28 MB TIF) [file pone.0005701.s004.tif]

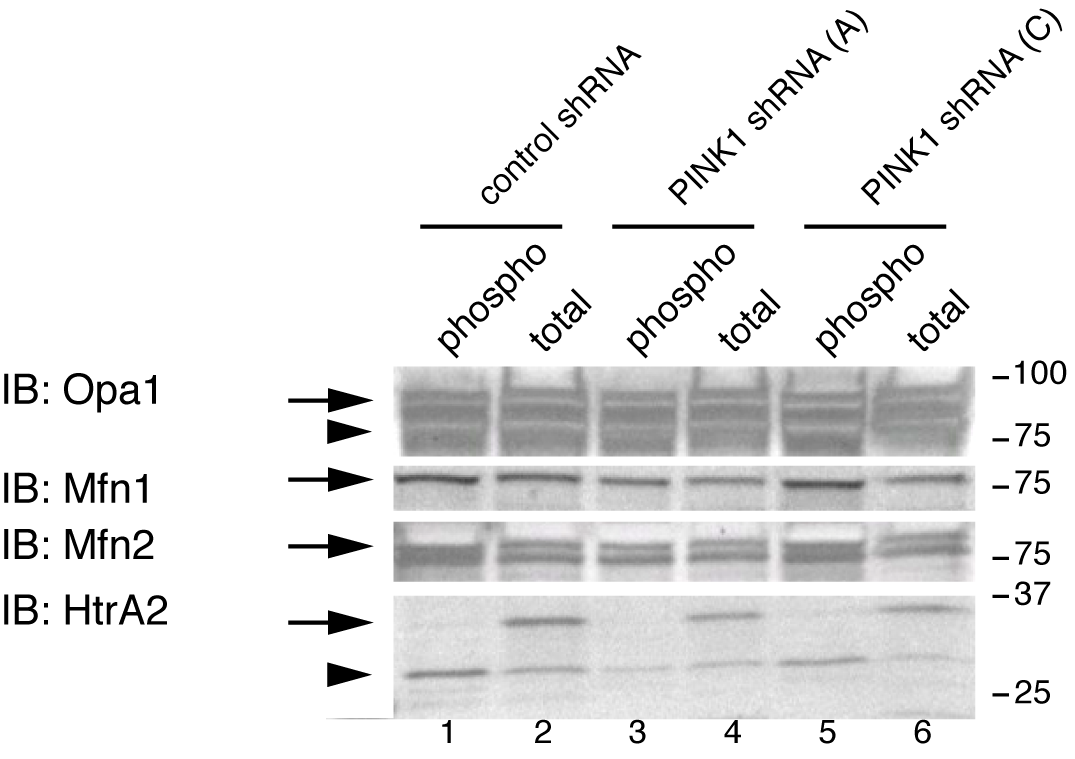

Supplement: Figure S5 — Analysis of additional proteins using phosphopurification in control and PINK1 shRNA cell lines To control for specificity in alterations of phospho-Drp1 in PINK1 knockdown cells, we examined the GTPases Opa1, Mfn1 and 2 and the protease Omi/HtrA2. Representative of triplicate independent experiments and purifications, no differences between cell lines were shown. Molecular weight markers are in kilodaltons. (0.34 MB TIF) [file pone.0005701.s005.tif]
